# Effects of green-wall layouts on psychological and physiological responses in office environments: a virtual-environment study

**DOI:** 10.3389/fpsyg.2025.1711317

**Published:** 2026-01-12

**Authors:** Yang Liu, Hengji Wang, Wenbo Li, Yihe Li

**Affiliations:** School of Art and Design, Zhejiang Sci-Tech University, Hangzhou, China

**Keywords:** green wall, EEG, virtual reality (VR), emotional well-being, office environment, fatigue recovery effect

## Abstract

**Introduction:**

Office greenery is closely related to employees’ physical and mental health. Green walls have been shown to provide biophilic and emotional health benefits; however, previous studies have mostly focused on the presence or absence of green walls or on their coverage area, and have rarely examined how their overall geometric morphology differentially affects users.

**Methods:**

In this study, we used virtual reality (VR) to construct four types of office scenarios, namely a curvilinear green wall (CGW), a linear green wall (LGW), a polyline green wall (PGW), and a no green wall condition (NGW), and evaluated their restorative effects after stress induction using MAST. On the psychological level, we adopted the Fatigue Scale FS-14 and the Restoration Outcome Scale ROS; on the physiological level, we recorded EEG data and used β∕α, (α + θ)∕β, θ∕β, and θ∕α to represent arousal, fatigue, attention, and relaxation, respectively, and calculated FAA to reflect motivational states.

**Results:**

The results showed that: (1) all three green-wall conditions were significantly superior to NGW in subjective ratings and EEG indicators, indicating that green walls can bring significant health benefits. (2) Regarding green-wall morphology, CGW was the most effective in reducing the fatigue index and reached a medium effect size compared with LGW; it also ranked first in enhancing attention and relaxation, although its differences from LGW did not reach statistical significance. This suggests that the curvilinear morphology further strengthens fatigue recovery while retaining the general advantages of green walls. (3) Compared with the other groups, PGW was overall disadvantaged across the EEG ratios; it only showed a small effect-size difference from CGW on arousal and achieved the highest median value. This indicates that the advantage of PGW lies mainly in enhancing arousal, whereas its other physiological performances do not reach the level of the more typical green-wall conditions.

**Conclusion:**

These findings indicate that, although all types of green walls are effective in promoting fatigue recovery compared with the no-green-wall group, their effects are not uniform; green-wall morphology can effectively modulate recovery effects and present differentiated advantages across different dimensions.

## 1 Introduction

As the service sector and knowledge-based economy develop rapidly and urbanization deepens, an increasing number of workers are shifting from physical labor to office-based mental work (Deng and Deng, 2018; Klitzman and Stellman, 1989; Klepeis et al., 2001). However, the stress and fatigue caused by high-intensity work not only lead to decreased productivity but also fundamentally undermine workers’ health status and increase morbidity (Marin et al., 2011; Awada et al., 2023; Bluyssen et al., 2011; Lewtas, 2007), for example through conditions such as sick building syndrome (Bluyssen et al., 2011). By contrast, exposure to outdoor natural environments can bring significant positive physiological and psychological health benefits, including relieving stress, enhancing cognitive performance, and improving emotional states (Hartig and Kahn, 2016; Yin J. et al., 2020; Kinnafick and Thøgersen-Ntoumani, 2014). In order to better integrate “green” into everyday living spaces, biophilic strategies that introduce natural elements into indoor environments have gradually become an important means of improving the quality of office environments (Tokuhiro et al., 2025; Jeong and Park, 2021).

To integrate plants more efficiently into everyday living environments, architects and landscape practitioners are combining vertical building surfaces with climbing/hydroponic planting systems to create sustainable vertical greening solutions (Grabowiecki et al., 2017; Perini and Rosasco, 2013). Previous studies have examined the environmental value of green walls from multiple perspectives, including green-wall types and structures (Manso and Castro-Gomes, 2015), air purification (Feng and Hewage, 2014), noise insulation (Wong et al., 2010), and carbon sequestration (Charoenkit and Yiemwattana, 2016). However, these studies primarily focus on the ecological functions, cultivation techniques, and other objective attributes of green walls and their derived environmental benefits, while empirical investigations into the health benefits of green walls through visual perception, such as alleviating psychological stress and promoting emotional restoration, remain relatively limited. Moreover, in biophilic design that affects human psychological and physiological responses, the dimensions of visual perception are not limited to the selection of plant species and material combinations; visual form features such as shape and contour are also important factors influencing individuals’ physiological states and psychological experiences (Tokuhiro et al., 2025; Tong and Shao, 2017). Such forms often carry specific perceptual cues and symbolic meanings and are closely associated with individuals’ visual preferences (Leder et al., 2011; Westerman et al., 2012; Bar and Neta, 2007; Reber et al., 2004). When combined with greenery, these forms influence people’s aesthetic preferences and psychophysiological perceptions (Tokuhiro et al., 2025; Gerstenberg and Hofmann, 2016). This means that, compared with single potted plants, green walls, as an artificially controllable plant-assembly medium, offer much richer possibilities for formal configuration. However, despite a large body of literature on the effects of plant visual characteristics, such as plant color (Wang et al., 2020; Jang et al., 2014), composition (Zhang et al., 2024; Shi et al., 2022), and size (Tokuhiro et al., 2025), existing research on the visual benefits of green walls still largely remains at relatively coarse levels, such as whether green walls are present and how much area they cover (Ma et al., 2024). There is a lack of systematic investigation into how the overall morphological configuration of green walls differentially affects relaxation, heightened alertness, and sustained attention.

In summary, existing studies have already examined in some depth the health effects of indoor green plants. However, although research on plants themselves and their visual characteristics is relatively abundant, most of this work still remains at the level of landscapes or natural vegetation, and it remains an open question whether green walls can likewise produce significant psychological and physiological restorative effects in indoor stress contexts. Moreover, morphological features often elicit individuals’ visual preferences and content perception, and it is unclear whether such form-driven processing differences can further influence the restorative effects of green walls. Building on these questions, the present study designs several common green-wall forms to examine whether form can serve as an important variable influencing the psychological and physiological effects of green walls, and further investigates on which specific physiological and psychological perception indicators these different forms are reflected.

## 2 Literature review

### 2.1 Regulatory effects of green walls on psychological and physiological responses under stress

Biophilic design has been shown to effectively improve building environmental conditions and enhance individual well-being (Yin J. et al., 2020), and its theoretical basis lies in the notion that humans have an innate preference for natural forms and living systems (Falk and Balling, 2010). Early studies used 2D images to explore the potential effects of biophilic elements on visual perception, and found that natural environmental scenes presented via 2D images could still increase perceived restorativeness scores to improve psychological states, as well as enhance EEG alpha activity, increase EMG amplitude, and reduce BVP to regulate people’s tension levels (Chang et al., 2008; Bartels and Zeki, 2004). In addition, the benefits of natural elements have also been corroborated in real-world studies by Serra et al., who found that landscapes designed with natural elements such as plants and water features, compared with landscapes without such arrangements, could effectively improve hospitalized children’s neuropsychological status, attention, and adaptability (Serra et al., 2025). With the development of virtual reality technology, VR has shown unique advantages in constructing highly controlled experimental environments that exclude external interference, which has led researchers to use it to explore the relationships between different environmental characteristics and psychophysiological responses (Kuliga et al., 2015; Mikropoulos and Natsis, 2011). This approach has likewise been applied to the analysis of biophilic elements; for example, some studies have shown that when a biophilic environment is constructed in virtual space, participants exhibit comparable levels of heart rate variability and parasympathetic nervous activity after exposure, indicating that biophilic designs constructed in virtual space can produce restorative effects that approximate those of real-world designs (Serra et al., 2025). Chen et al. designed a VR experiment and showed that participants exposed to an indoor environment featuring nature-themed artworks did not differ significantly from those experiencing natural window views in terms of recovery effects on systolic blood pressure, heart rate, and other indicators, and that both conditions produced more pronounced reductions in physiological stress and greater relaxation than an architectural-window baseline condition (Chen et al., 2025). Therefore, using virtual environments to examine the effects of biophilic variables on psychological and physiological responses has become a relatively mature and feasible approach.

As a design element commonly used on building facades and in indoor spaces, green walls have been shown to fully leverage the advantages of biophilic design and exert positive effects on multiple physiological indicators, including cardiovascular responses (heart rate, blood pressure, and heart rate variability), skin conductance, and EEG activity (Yeom et al., 2021; Brown et al., 2013; Van den Berg et al., 2015). Specifically, compared with indoor spaces without green walls, visitors in environments with green walls show significantly lower systolic blood pressure and heart rate, and even brief viewing of green walls can increase heart rate variability and buffer the decline in heart rate variability induced by stress tasks (Shao et al., 2024; Ma et al., 2024). At the level of longer-term effects, green walls in indoor natural environments can markedly reduce individuals’ subjective anxiety and tension, while effectively increasing alpha and beta activity in physiological EEG (Jeong and Park, 2021). Moreover, the restorative effects of green walls can still be elicited in virtual settings. Yeom et al. (2021), using virtual reality simulations, showed that in underground spaces the presence of green walls can effectively accelerate cognitive restoration and simultaneously increase bodily thermal comfort with respect to the environment (Ma et al., 2024). Taken together, this body of research indicates that, compared with single potted plants, composite visual arrangements of plants tend to be more advantageous in terms of immediate visual impressions and long-term emotional benefits (Dzhambov et al., 2021; Larsen et al., 1998; Lindemann-Matthies and Matthies, 2018). However, few studies have examined the post-stressor restorative effects of whole green-wall systems across multiple physiological and psychological levels. Most research has focused on the restorative role of single plants or local visual features, or has only tested the effects of green walls on a limited set of indicators symbolizing relaxation, and has not yet systematically investigated the restorative mechanisms and differentiated effects of green walls as an integrated system.

### 2.2 Effects of green-wall morphology on psychophysiological responses

In the studies by Armbruster et al. (2014), object shape was found to exert specific effects on the autonomic nervous system (ANS) and on individuals’ emotional states (Blazhenkova and Kumar, 2018). This evidence provides a feasible basis for further exploring the mechanisms through which plants affect people under conditions where color and area are held constant. Existing research has also indicated that, in terms of aesthetics, humans tend to prefer targets that can be rapidly recognized by the brain and processed with high fluency (Hůla and Flegr, 2016). For example, when the overall contour more closely resembles curves and natural forms, it is more likely to evoke associations with plant-like shapes, receive higher subjective visual preference ratings, and effectively alleviate subjective fatigue (Tawil et al., 2022; Gómez-Puerto et al., 2016). This perceptual preference for form is likewise reflected in the plants themselves. Empirical studies have shown that plant-related visual features such as morphology and composition can modulate place-related feelings including perceived safety and a sense of oppression (Hareli et al., 2016; Lis, 2025; Lis and Iwankowski, 2021), thereby reshaping individuals’ overall experience of space at the level of perception. Moreover, such form-related effects or preferences also emerge at the level of whole or grouped plant compositions. For instance, in indoor environments individuals typically prefer plants that are smaller in size, compact in posture, or spherical in form, whereas tall plants with broad leaves are often regarded as the least favored type (Tokuhiro et al., 2025). In more fine-grained studies on morphological effects, Tong and Shao (2017) showed that plants pruned into geometric forms and plants allowed to grow naturally produce significantly different physiological effects: participants who experienced naturally shaped plants exhibited a significant decrease in average diastolic blood pressure accompanied by more pleasant emotional experiences, whereas those who viewed geometrically shaped plants did not show the same magnitude of blood pressure reduction but displayed a more stable and harmonious overall emotional state. However, this line of research mostly takes “plant form” itself as the starting point and focuses on the visual and physiological effects of single plants or plant groupings, and relatively few studies examine, from the perspective of the green wall as an overall interface, the impacts brought about by differences in form. In recent research, Li et al. (2025) explicitly treated “morphology” as an independent variable and examined the differentiated restorative effects of three geometric green-wall forms (rectangular, square, and triangular) on subjective evaluations and physiological indicators. The results showed that rectangular green walls (including rectangles and squares) were more conducive to reducing cognitive load and promoting attention restoration, whereas triangular green walls required individuals to invest more cognitive resources and maintain higher alertness, and thus were not suitable as a morphology for relaxing environments (Li et al., 2025). These studies not only confirmed the facilitative effects of plants using both physiological and psychological measures, but also indicated that when “morphology” is explicitly used as an independent variable, it can shape, to some extent, the direction and preferential pathways of health benefits.

Overall, previous research has mainly focused on “plant morphology itself” and its visual effects, while discussion of green-wall morphology has mostly remained at relatively coarse definitions such as the presence or absence of green walls or the size of rectangular configurations assembled from modules (Ma et al., 2024), and there is still a lack of studies that systematically examine how differences in overall structural morphology lead to differentiated effects on relaxation, alertness, and attention after stress. On this basis, it is therefore particularly important to investigate the visual utility of green-wall morphology and further explore its potential impact on psychophysiological responses during the process of stress recovery.

### 2.3 Research objectives and hypotheses

Research objective 1: Compared with ordinary indoor environments, biophilic design has been shown by relevant studies to exhibit significant advantages in both subjective experience and multiple physiological indicators, including heart rate, blood pressure, heart rate variability, skin conductance responses, and EEG frequency-band characteristics related to relaxation and arousal. However, most of this evidence comes from general green-environment settings, such as outdoor landscapes with plants or indoor potted plants, and there is a relative lack of studies that systematically test their “restorative effects” under clearly defined stress conditions, with many relying on only a limited set of single indicators for evaluation. To address this gap, the present study proposes the following hypotheses:

*H1*: Compared with the no green wall condition, all green wall conditions will produce more positive psychological and physiological restorative effects.

*H1a*: In terms of psychological restoration, participants who experience conditions with green walls will show more positive psychological recovery and lower levels of perceived fatigue.

*H1b*: In terms of physiological restoration, compared with the no green wall condition, participants who experience green wall conditions will exhibit more favorable performance on physiological indicators, including fatigue level, arousal index, attentional control index, and relaxation.

Research objective 2: Building on existing studies showing that plants and their morphological features can influence individuals’ subjective preferences as well as psychophysiological responses such as blood pressure, heart rate, and fatigue, this study further focuses on exploring the differential effects of green-wall morphology (curvilinear, polyline, and linear). The aforementioned literature suggests that curvilinear forms that are more natural and contour-continuous are more likely to elicit subjective preference, reduce fatigue, and promote restoration; triangular or polyline forms with sharp angles are more likely to heighten vigilance and arousal; and regular linear forms help create a sense of order, thereby supporting relaxation experiences and the maintenance of attention. However, related research on these three types of forms has not yet been systematically examined using green walls as a unified medium. On this basis, the present study proposes the following hypotheses:

*H2*: As morphology varies, different green-wall forms will show differences in their physiological and psychological restorative effects.

*H2a*: At the subjective level, the curvilinear green wall will elicit more favorable outcomes in terms of fatigue relief and perceived restoration than the other green-wall forms.

*H2b*: In terms of physiological indicators, the curvilinear green wall will lead to more beneficial fatigue recovery and more positive affect than the other green-wall groups.

*H2c*: Regarding physiological indices, the polyline green wall will produce higher arousal levels than the other green-wall groups.

*H2d*: With respect to physiological indicators, the linear green wall will yield more favorable relaxation effects and attentional control than the other green-wall groups.

## 3 Method

### 3.1 Participants

Given that this study involved EEG acquisition, males and females may differ in scalp thickness and certain physiological characteristics, which could in turn affect signal quality and the stability of results (Jaušovec and Jaušovec, 2010). To reduce bias introduced by sex-related differences, we strictly controlled the sex ratio during recruitment and achieved a balanced allocation of males and females across experimental groups. Accordingly, a total of 174 volunteers (87 males, 87 females) were enrolled via on-site recruitment and email invitations. After data collection, 14 participants were excluded due to poor recording quality identified during preprocessing that could compromise the analysis. The final sample comprised 160 volunteers, 80 males and 80 females, aged 18–45 years, with a mean body mass index of 21.6 kg/m^2^. Before formally taking part in the experiment, all participants were required to meet the following inclusion criteria to ensure accurate EEG recording and stable wearing of the virtual reality (VR) equipment: (1) no history of cardiovascular disease, neurological disorders, or other severe chronic illnesses, and no habits such as smoking or drinking that might interfere with physiological measurements; (2) self-reported normal vision or vision corrected to normal, and confirmation of no red–green color blindness or red–green weakness via a color-vision screening using 24 color-plate tests; (3) adequate sleep within the 24 h preceding the experiment and avoidance of alcoholic or caffeinated beverages. A single-blind design was adopted: participants were informed only that the experiment aimed to assess individuals’ perceptual experiences of environments, and the link between the experimental materials, EEG signals, and subjective questionnaires was kept strictly confidential to avoid expectancy effects on the data. Prior to the formal experiment, all participants signed a written informed consent form approved by the Institutional Review Board. The consent form detailed the experimental procedures, potential risks, privacy protections, and data anonymization protocol, thereby safeguarding participants’ right to be informed and their ethical interests. Upon completing the experiment, each participant received a standardized thank-you gift valued at 70 RMB (approximately USD 10) as compensation for their time and cooperation. The entire procedure of this study strictly followed the ethical principles involving human participants as stipulated in the Declaration of Helsinki (DoH), ensuring that the research conformed to international standards in both scientific rigor and ethical conduct.

### 3.2 Laboratory environment

**Figure 1:**
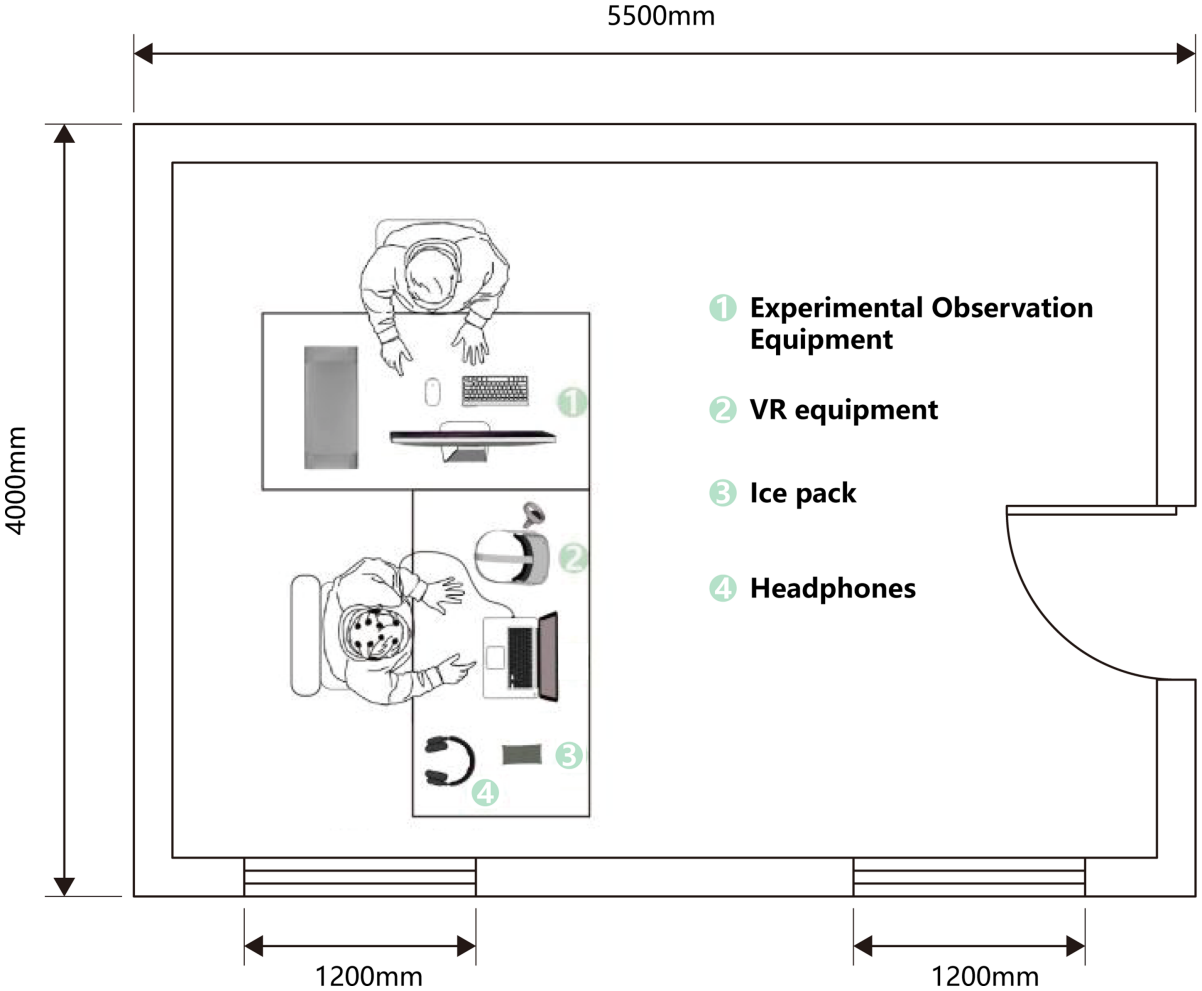
Floor plan of the laboratory site.

Prior studies have demonstrated that various indoor environmental quality (IEQ) factors, such as air temperature (°C), relative humidity (%), CO₂ concentration (ppm), and illuminance (lx), can influence people’s psychological and physiological responses (Geng et al., 2017; Deng et al., 2024). Accordingly, the present study controlled all external variables other than the visual effects of green walls to prevent them from becoming confounders. The experiment was conducted in an artificial climate chamber at a university in Hangzhou (location: 30.478° N, 120.289° E; subtropical monsoon climate). The experimental space was a standard rectangular laboratory (length 4.0 m, width 5 + 0.5 m, height 3.2 m) with neutral white walls and diffuse ceiling lighting. Air temperature was maintained at 28 ± 0.5 °C, relative humidity at 40 ± 10%, illuminance uniformly at 300 ± 50 lx, and CO₂ concentration between 350 and 450 ppm. The experiment lasted 60 days, with two daily sessions (09:00–11:30 and 14:00–16:00). During the experiment, participants wore a VR headset and could freely explore the virtual scenes using a 360° swivel chair or handheld controller. Notices were posted outside the laboratory to restrict access and prevent nonparticipants from generating external noise, as shown in Figure 1.

### 3.3 VR-based design of experimental materials

The VR not only facilitates the reconstruction of scenarios under controlled laboratory conditions, thereby effectively reducing site-related complexity and potential confounders, but also offers strong operability and reusability (Newman et al., 2022; Yin J. et al., 2020; Spano et al., 2023). Consequently, VR technology has been widely and maturely applied across domains such as gaming, education, tourism, and architecture (Tian et al., 2021). Moreover, multiple studies have used VR to examine the effects of green walls on physiological responses including EEG, electrodermal activity, and heart rate (Yeom et al., 2021; Yin et al., 2019; Gao et al., 2019), providing a solid theoretical and methodological foundation for the present study’s procedure and material selection.

#### 3.3.1 Office environment design

**Figure 2:**
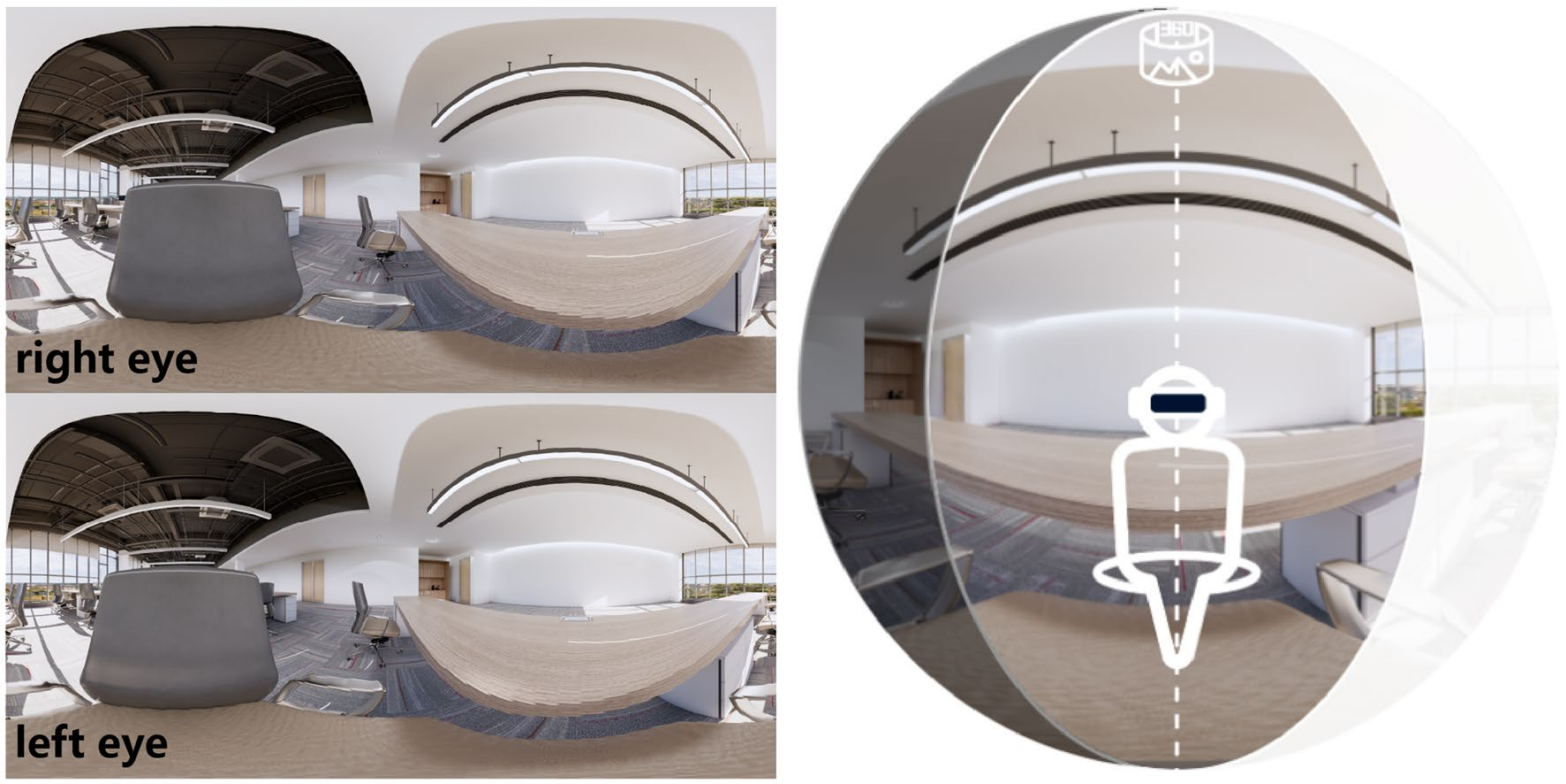
360° three-dimensional equidistant projection example (left image) and schematic diagram of participants immersed in this scene (right image).

In this study, SketchUp 2023 was used to construct the three-dimensional model of the site, and Enscape Custom Asset Editor was employed to render and capture stereoscopic views, generating 360° panoramic images. Given that the study focuses on fatigue effects in office settings, the time was set to 8:30 a.m., which corresponds to the typical start time of morning shifts in this region of China. At this time of day, the outdoor solar azimuth angle is 101.2°, and the solar altitude angle is 27.1°. All indoor observation points were set from the seated viewpoint of adult participants, with the camera height fixed at 1.25 m. Participants were positioned facing the green wall, which was placed at a distance of 4 m from the seating position in the site model. The construction of the experimental model adhered to these requirements, as shown in Figure 2.

**Figure 3:**
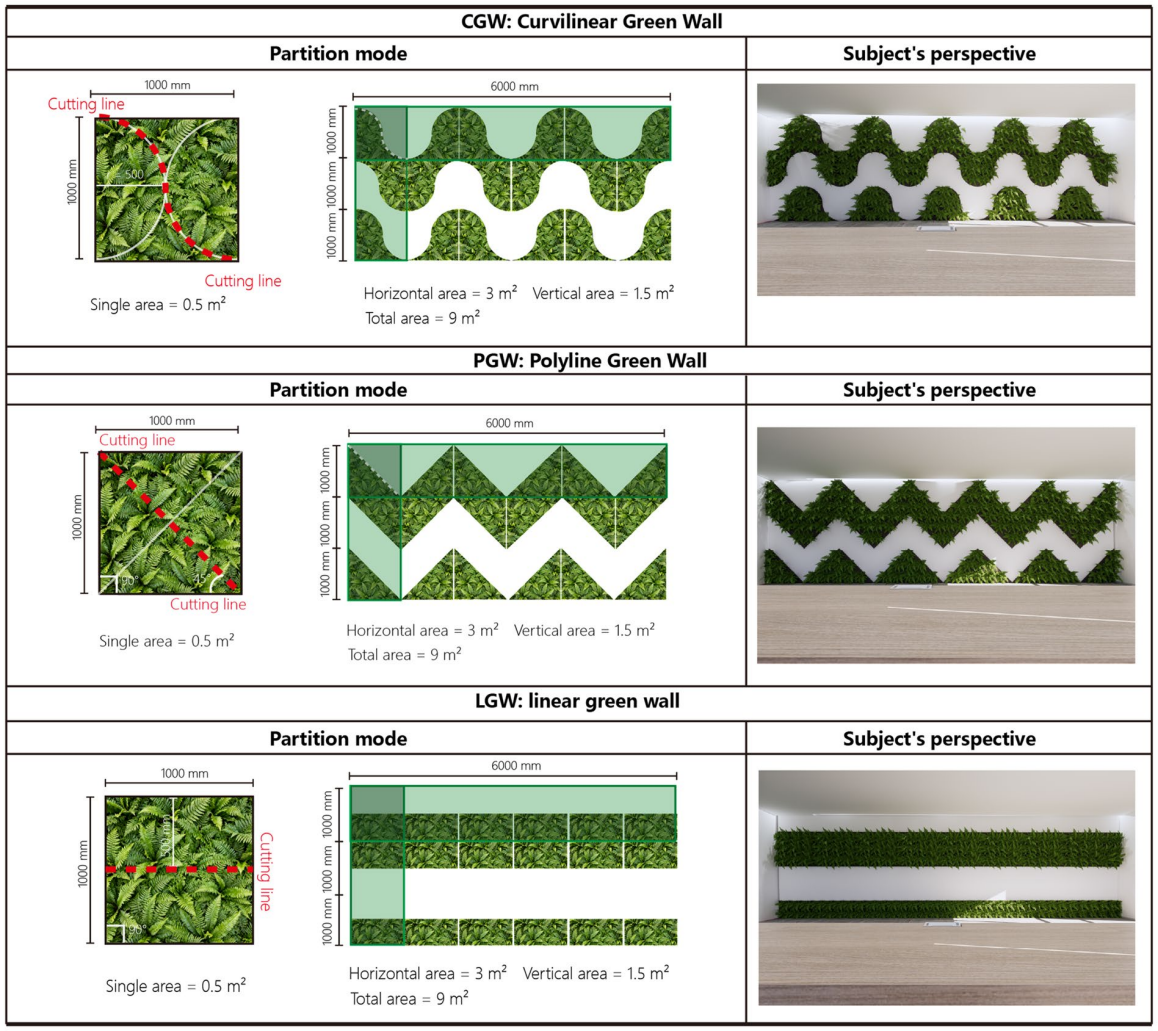
Modular geometric configurations of green walls and participants’ VR viewpoints.

The VR device used in this study was the Oculus Quest 3. The device has a maximum brightness of 80 cd∕m^2^, a field of view of 90°, a monocular resolution of 1832 × 1920 and a binocular resolution of 3,664 × 1920, with a refresh rate of 90 Hz. Its interaction modes provide two options: free movement and fixed position. In the pilot experiment, a room-scale free-movement mode was used so that participants could explore the space in advance before the formal experiment; after the formal session began, the mode was switched to a fixed-view mode to eliminate potential interference of viewpoint shifts on the measurements, and an example of the participant’s view is shown in Figure 3.

#### 3.3.2 Green-wall materials design

The green wall was designed as a modular installation, an application based on lightweight, permeable mesh into which plants are inserted individually; therefore, the size of plants used in each planting module must be considered (Manso and Castro-Gomes, 2015). We selected Boston fern as the plant species for this experiment, not only because its growth requirements suit our locale, but also because participants were recruited locally and this choice better accords with local aesthetic preferences (Goswami et al., 2016; Nitta et al., 2022; Oloyede, 2012).

In terms of layout, we adopted modular combinations of geometric patterns, because they are low-cost, easy to construct, low in energy consumption, convenient for module-based design and expansion, and easier to implement in real-world settings (Manso and Castro-Gomes, 2015; Shushunova et al., 2023). Using these geometric modules, we assembled three green-wall morphological designs, namely curvilinear, polyline, and linear. Meanwhile, the linear green wall, as a commonly used application form, served as a reference to examine whether morphological design introduces additional effects. To ensure that the different layouts differed only in shape, all three green walls were derived by cutting a square green wall of equal area into fixed geometric units and then recombining them with the same total area. As shown in Figure 3, the wall was divided into six vertical by three horizontal units, and the area proportion of each unit was kept identical so that comparisons would not be confounded by differences in greening area or coverage ratio. Finally, the experimental materials were divided into four groups according to plant layout: three experimental groups, namely the curvilinear green wall (CGW), the polyline green wall (LGW), and the linear green wall (PGW), and one no–green wall office (NGW) as the control group.

### 3.4 Stressor design

Because this study aimed to evaluate fatigue and restorative effects, it was necessary to induce mental fatigue prior to the formal measurements (Zhang et al., 2023). To this end, we adopted the simplified Maastricht Acute Stress Test (MAST) as a standardized stress paradigm. This paradigm is easy to administer and low in cost, and the psychological and physiological responses it elicits have been empirically shown to resemble those of real-world stressors. MAST comprises two sequential stressors: a cold-pressor task and mental arithmetic. The cold-pressor phase lasted 30 s, during which participants placed both palms in full contact with an ice pack to induce painful physiological stress. The mental-arithmetic phase lasted 90 s, during which participants performed serial subtraction with a four-digit number while speaking the answers aloud (e.g., “2013 minus 17 repeatedly”); upon any spoken error, they restarted from the beginning. Under the combined effects of tension cues and time pressure, this procedure induces psychological stress (Liu et al., 2024). To ensure stress intensity and stability, the above sequence was repeated twice. Experimental control was implemented with PsychoPy v3.0.

To further increase cognitive load and ecological validity, a noise stimulus was superimposed during the stress phase. Construction noise was selected because it can increase mental workload and amplify the stress experience (Ke et al., 2021). The noise intervention was processed using Adobe Audition 2025, with the sound pressure level strictly controlled at 75 dBA to match the moderate exposure commonly experienced by construction workers and to align with the effective stress intensity employed in related studies (Kawai et al., 2024). Audio playback used Beyerdynamic DT 770 Pro studio headphones to ensure consistent isolation performance (Isnard et al., 2024). Based on the conversion between headphone sensitivity and rated power, the sound was maintained at 75 dBA throughout the experiment to ensure that the stimulation intensity remained constant and reasonable, while also balancing safety, realism, and experimental validity.

### 3.5 Measurement methods

#### 3.5.1 Subjective psychological measures

We used two established and well-validated questionnaires: a fatigue scale (Chalder et al., 1993; Zhang et al., 2023) and a restorative outcomes scale (Suko and Korpela, 2024; Takayama et al., 2014).

Fatigue severity was assessed using the FS-14. Participants first completed the FS-14 questionnaire (Appendix Table 1). This scale evaluates the validity and intensity of the fatigue process and comprises 14 items, including negatively worded statements such as “Do you feel fatigued?,” which effectively capture negative experiences. Each item was rated on a seven-point Likert scale from 1 (not at all) to 7 (strongly agree). To facilitate comprehension and situational focus, we employed a localized translation and added the introductory cue “While experiencing this space (or afterwards), I feel…” at the beginning of the questionnaire to guide participants to attend to stress changes during the experimental phase rather than to general bodily sensations (Beaton et al., 2000). Given that all FS-14 items are negatively worded, responses were reverse-scored during data analysis and then aligned with the restorative scale results to enable integrated comparisons.

The Restorative Outcome Scale (ROS) was used to assess affective and cognitive recovery in specific environments, and was developed based on existing research and measurement frameworks on restorative experience. The scale contains six items: three reflect relaxation and calmness, one reflects attention restoration, and two reflect mental clearing. The ROS is rated on a seven-point Likert scale from 1 (not at all) to 7 (strongly agree). Overall, the ROS captures individuals’ short-term restorative experiences within the target environment and facilitates comparative analyses with physiological indicators (Korpela et al., 2008; Staats et al., 2003) (see Appendix Table 1 for details).

Prior to the formal experiment, we conducted a pilot test with 30 participants using the three experimental green-wall conditions (CGW, PGW, and LGW). After exposure, participants completed the questionnaires, and we performed exploratory factor analysis and confirmatory factor analysis to verify reliability. Cronbach’s α was 0.871 for the ROS and 0.822 for the FS-14, both exceeding the criteria reported by Gliem and Gliem (2003). In the formal experiment, Cronbach’s α for the ROS ranged from 0.823 to 0.862 across the four groups, and for the FS-14 ranged from 0.822 to 0.876—meeting common standards for internal consistency.

#### 3.5.2 Objective physiological measures

This experiment selected electroencephalography (EEG) as the physiological indicator. Owing to its high temporal resolution and objectivity, EEG is widely used for dynamic monitoring of physiological responses (Li et al., 2022; Reis et al., 2014). EEG records the electrical activity of populations of cortical neurons; by examining this activity, one can infer the participant’s current state, such as wakefulness, sleep, concentration, and relaxation (Song and Tagliazucchi, 2020; Coenen, 1995; Blinowska and Durka, 2006), and it has already been employed extensively in literature on plants’ physiological effects on humans (Yeom et al., 2021; Pei et al., 2025; Ding et al., 2022; Jang et al., 2014).

Emotion is a complex construct comprising multiple dimensions such as drowsiness, relaxation, and alertness. Relying on a single indicator (e.g., FAA) often fails to capture the subtle psycho–physiological differences elicited by different green-wall morphologies (Kim et al., 2017). Moreover, EEG power itself is prone to substantial inter-individual variability, for example due to scalp/soft-tissue thickness (Cuffin, 2002), age structure and aging processes (Carrier et al., 2001; Tröndle et al., 2023), and health status or medication history (Morgan et al., 2005), making absolute values in a single band an unstable representation of true physiological state. On this basis, we compared band-power ratios such as (α + θ)/β, β/α, θ/β, and θ/α (Xiao et al., 2024; Eoh et al., 2005). By integrating relative changes across two or more bands, these indices offer broader information coverage and can, to some extent, offset individual differences and external interference affecting single-band power, thereby providing a more robust representation of participants’ current states (Freeman et al., 1999). Their indicative meanings are as follows:

The β/α ratio has been recognized in prior research as an effective index for visual evaluation (Hsu and Wang, 2013). In the EEG data collected here, the β/α ratio was used to index arousal level (Suh and Yim, 2018); higher β/α results indicate stronger task engagement and involvement (Kislov et al., 2022). Meanwhile, the (α + θ)/β ratio is commonly used to characterize fatigue level or the activation state of participants (Eoh et al., 2005). Some studies investigating the relationship between physiological parameters and driver status have found that as driving time increases, the (α + θ)/β ratio gradually rises, indicating increasing fatigue, while the β/α ratio gradually declines, reflecting reduced arousal (Xiao et al., 2024).

In addition, the θ/β ratio was first used as an EEG index for diagnosing attention-deficit/hyperactivity disorder (ADHD) and for assessing attentional control; subsequent studies in healthy populations have shown that θ/β is negatively correlated with self-reported trait attentional control (Lubar, 1991; Brookhuis and De Waard, 1993; Putman et al., 2014). The alpha/theta protocol is known as a classic neurofeedback method for stress reduction and for inducing an increase in the θ/α rate; in the study by Matousek and Petersén (1983), the θ/α ratio was shown to decrease when participants were in a relaxed state (Chen et al., 2013; Strijkstra et al., 2003).

Frontal alpha asymmetry (FAA) refers to an unequal distribution of wave power between the left and right cerebral hemispheres, primarily observed in the frontal regions. FAA is typically assessed by measuring alpha activity in the left and right frontal cortices, because alpha waves are the most prominent rhythm in the brain, especially in the frontal areas. Alpha activity is associated with emotion regulation and motivational systems, and its power is generally regarded as an inverse index of cortical activation, meaning that lower alpha power in a given region indicates stronger cortical activation in that region. In previous studies, FAA has been widely used to evaluate the effects of nature-based interventions and environmental scene design on individuals’ emotional states (Ma et al., 2024), and beyond emotional evaluation, research by Harmon-Jones et al. (2010) has shown that FAA is closely related to motivational direction, such as approach versus withdrawal, rather than merely reflecting emotional valence. Therefore, in this study FAA is regarded as a neurophysiological indicator of environmental motivation under different green-wall morphologies, used to complement the subjective fatigue and restoration scales, and to examine at the motivational level whether different green-wall forms can elicit stronger environmental approach tendencies and restorative experiences.

#### 3.5.3 Physiological data collection tools

EEG data were acquired using the Emotiv EPOC X portable electroencephalography system, which has been widely applied in VR-based neurocognitive research (e.g., attention, emotion regulation) and in quantitative assessments of how environmental quality affects psychophysiological responses (Castiblanco Jimenez et al., 2024; Krogmeier et al., 2022). The device features 14 EEG recording channels and two reference electrodes (CMS/DRL). The built-in sampling rate is 2048 Hz, with a resolution of LSB = 0.51 μV (14-bit mode) or 0.1275 μV (16-bit mode). For data storage, signals collected from the 14 electrodes can be transmitted via Bluetooth 5.0 to a computer hard drive and to the product’s cloud software, emotivPRO.

### 3.6 Experimental procedure

**Figure 4:**
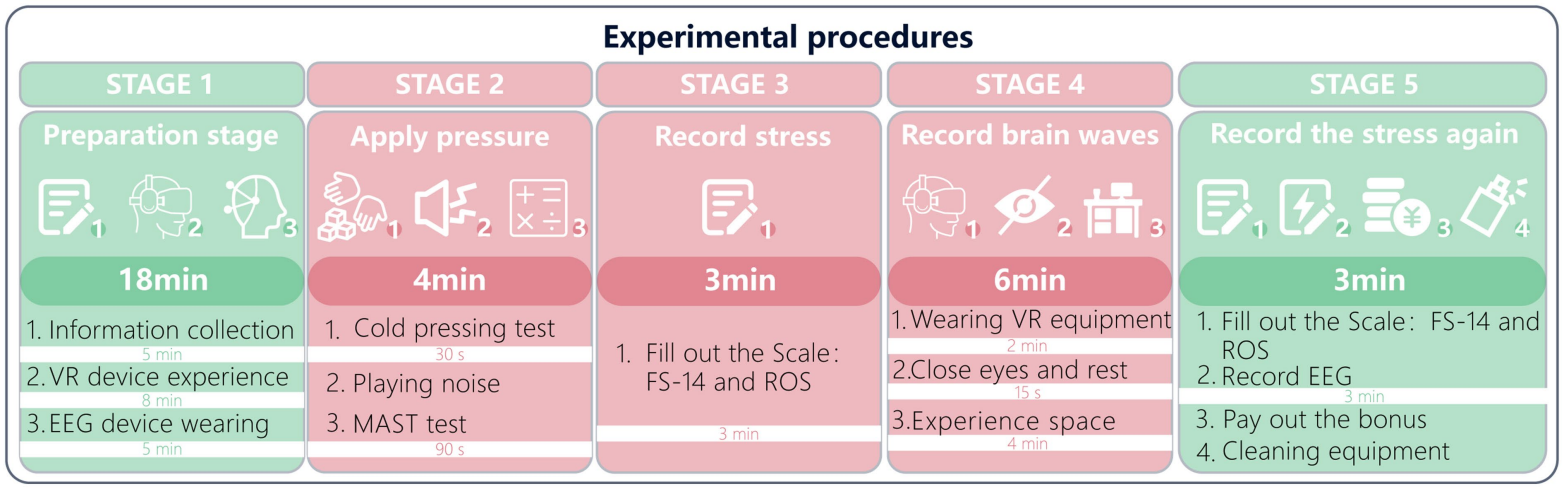
Experimental procedure of the present study.

Each participant’s session lasted about 35 min. The experiment consisted of five phases, as shown in Figure 4:

(1) Phase 1 (total duration about 18 min): This phase was used for explaining the experimental briefing and adjusting comfort. The researcher spent 5 min in the waiting room explaining the experiment to participants, collecting relevant information, and issuing the informed consent approved by the ethics committee. Participants were informed that they must wash their hair 1 h before coming to the laboratory. During the experiment, in order to reduce EEG noise, participants were asked to minimize swallowing, blinking, and eye movements as much as possible, without being forced to prohibit them; the upper body, especially the head, needed to remain comfortably stable (Welke and Vessel, 2022; Yin L. et al., 2020; Zhang et al., 2023). After confirmation, participants were escorted to the laboratory. We provided the VR device for a trial fitting and seated rest so that they could adapt to the virtual scenes in STEAM VR; they could adjust the VR device, chair height, and comfort according to their own situation. This stage lasted 8 min. After the trial fitting, the researcher fitted the EEG device and performed debugging, ensuring that all sensor pads were moistened with saline to optimize scalp conductivity, and checked that EEG signals were being recorded normally; this stage lasted about 5 min.

(2) Phase 2 (total duration about 4 min):this phase mainly uses testing to stimulate participants so as to increase their levels of stress and fatigue. Accordingly, participants are required to complete the MAST stress test, and before the formal test begins, the experimenter will guide them to understand the test procedures to ensure smooth administration, which may take about 1.5 min. After this introduction, the experimenter will first provide the participant with an ice pack, and the participant will be asked to place both palms on the ice pack and hold it for 30 s. Subsequently, the participant will perform a mental arithmetic task for 90 s while exposed to a noise audio track played by the experimenter.

(3) Phase 3 (total duration about 3 min): this phase is mainly used to record participants’ subjective stress and fatigue states. We will administer the compiled FS-14, ROS, and questionnaire (20 items in total) for participants to complete, in order to document their current subjective stress status.

(4) Phase 4 (total duration about 6 min): This phase mainly allowed participants to experience the office space with green walls, so as to observe the effects on stress and fatigue recovery during this stage. The experimenter spent about 2 min fitting the VR device for them. After the device was fitted, participants were placed within the VR device in one of the four experimental-material spaces we designed; the experimental material they experienced was randomized. The researcher could view the participants’ experience status on the computer and informed them that they could rotate the swivel chair or operate the controller to choose a preferred viewpoint and then remain relatively still; these actions enabled us to use standard cleaning procedures to remove most artifacts from the raw EEG data. Afterwards, participants were asked to close their eyes for 15 s to ensure ocular comfort and to facilitate EEG recording. After 15 s, the duration of experiencing the space was about 4 min, during which the researcher observed and recorded the participants’ EEG data, ensuring that the participants’ and device’s status met experimental standards.

(5) Phase 5 (total duration about 3 min): After the experience, participants were required to complete the FS-14 and ROS again to record their subjective feelings, and to finish within 3 min. Each participant’s pre- and post-exposure questionnaires were recorded in the same file to facilitate subsequent comparison. Finally, after ensuring that the participants’ EEG and questionnaire information had been comprehensively collected, the researcher helped remove the equipment and cleaned it to ensure subsequent use. Each participant who completed the session received a stipend of 70 RMB as experimental compensation.

### 3.7 Data processing

#### 3.7.1 EEG signal preprocessing

**Figure 5:**
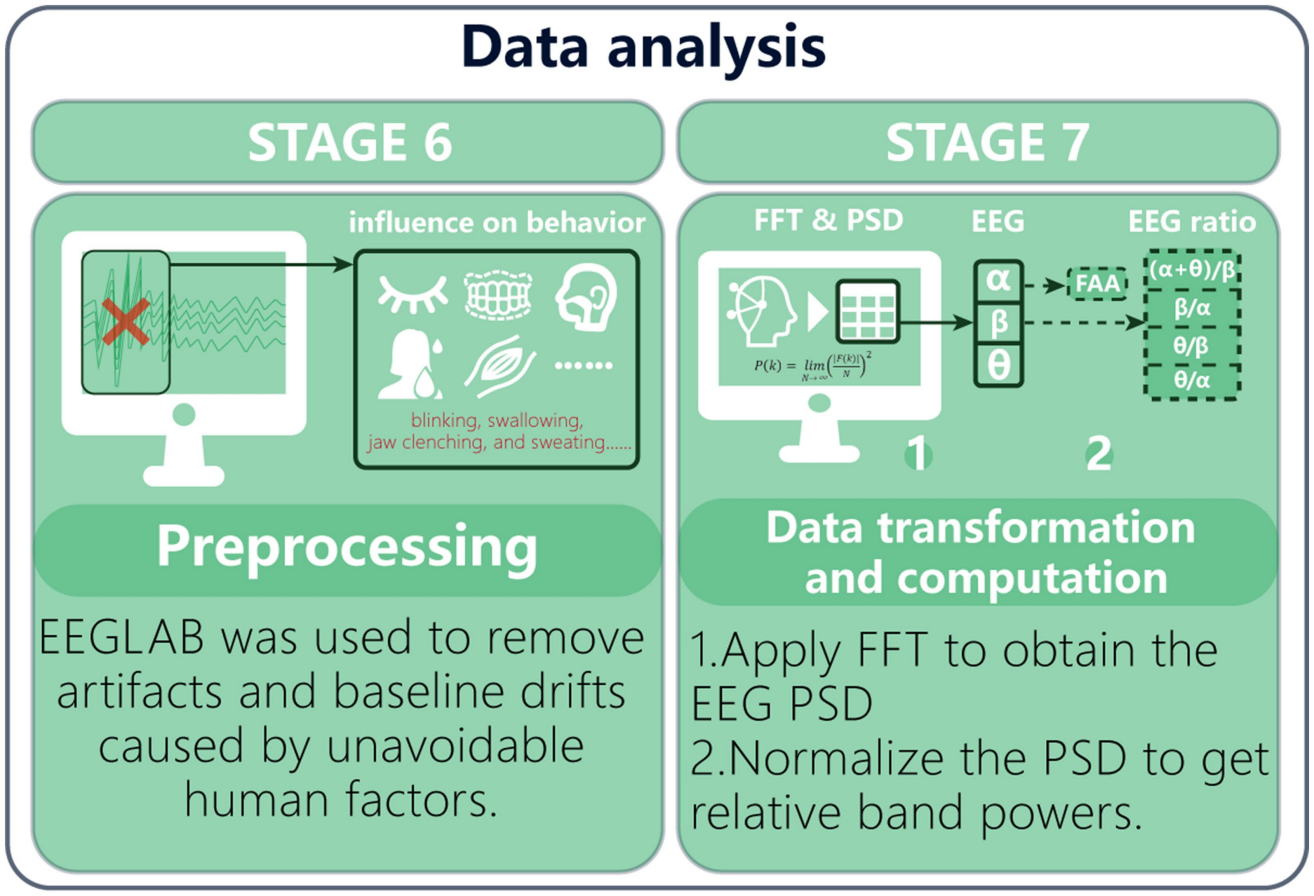
The preprocessing procedure of EEG.

The EEG signals are easily contaminated by physiological artifacts such as involuntary eye movements, blinks, cardiac activity, and electromyographic activity, which are difficult to eliminate completely and may affect the experimental conclusions (Jiang et al., 2019). To enhance the reliability of feature extraction from EEG, this study adopted a systematic preprocessing pipeline using EEGLAB on the MATLAB R2024b platform, and the data preprocessing steps are illustrated in Figure 5. We first trimmed the initial 30 s of EEG recordings (which included participants’ eyes-closed rest and effects of experimental prompts) and removed nonessential electrodes (e.g., HEOG and VEOG). After this step, a dynamic-threshold algorithm was used to identify and exclude channels with abnormal amplitudes (peak-to-peak > 500 μV), thereby ensuring the integrity of the acquisition system. In accordance with the technical specifications of the Emotiv EPOC X, a CMS/DRL active reference system was adopted in place of the traditional mastoid reference to reduce common-mode noise. Filtering was based on the study’s EEG frequency range of 1–30 Hz; specifically, a high-pass filter removed baseline drift below 1 Hz, and a low-pass filter removed frequencies above 30 Hz to suppress high-frequency EMG interference. In addition, a notch filter was applied to remove 50-Hz line noise. Subsequently, we applied independent component analysis (ICA) combined with the ADJUST automatic detection algorithm to identify and label noise components based on spatial distributions and temporal characteristics (e.g., ocular artifacts, muscular artifacts, and other non-neural activities), and these components were rejected. Two researchers independently inspected the data according to criteria such as signal fluctuation range < ±50 μV and residual-noise thresholds. Interrater agreement was evaluated with Cohen’s kappa (*K* = 0.82).

After completing conventional preprocessing of time-domain EEG signals, we computed frequency-domain spectra via fast Fourier transform (FFT) and calculated the power spectral density (PSD) for each channel. As a representation of energy distribution in the frequency domain, the PSD enables precise quantification of rhythmic power within specific oscillatory bands by analyzing the energy proportions across EEG frequency ranges (Pan et al., 2023). Equation (1) is the calculation formula for the Fourier transform, and Equation (2) is used to compute the power spectrum based on the results of the Fourier transform:


$$F[k]=\sum_{n=0}^{N-1}x[n]e^{-j\frac{2\mathrm{\pi kn}}{N}},\;\;k=0,1,\ldots ,N-1$$ (1)

$$P(k)=\lim_{N\to \infty }(\frac{|F(k)|}{N})2$$ (2)

In power spectral analysis, a normalized power spectrum (also called relative power) was used to reduce between-subject variability. Relative power values were computed for the four bands based on the frequency ranges of δ, θ, α, and β. This procedure was performed across the 14 EEG channels—calculating the relative power of the four bands for each channel. The calculation method of the normalized power spectrum is shown in Equation (3) and (4) [90]:


$$P_{\mathrm{norm},i,k}=\frac{P_{i,k}}{P_{\text{total},i}}=\frac{P_{i,k}}{\sum_{m\in \{\delta ,\theta ,\alpha ,\beta \}}P_{i,m}}$$ (3)

*P*_i,k_ denotes the absolute power of frequency band *k* at channel *i*, and *P*_total_ denotes the total power of that channel across the full frequency range. After normalization, the following conditions hold (Lang et al., 2022):


$$\sum_{k=1}^{4}P_{\mathrm{norm},i,k}=1$$ (4)

For this equation, the index *k* = 1, 2, 3, and 4 corresponds to the δ, θ, α, and β frequency bands, respectively. This processing ensures that power values across different participants and channels are comparable, thereby providing standardized inputs for subsequent statistical analyses.

For the calculation of FAA, four left-frontal channels (AF3, F7, F3, and FC5) and the corresponding four right-frontal channels (AF4, F8, F4, and FC6) were selected, and the power spectral density in the α\alphaα band was computed for these eight channels. The logarithmic difference was taken as FAA. Because α\alphaα-band activity is inversely related to the activation of the corresponding cortical regions, a larger FAA value indicates greater left-frontal activation relative to the right and thus a higher level of positive affect (Kelley et al., 2017; Ma et al., 2024). The calculation process of FAA is shown in Equation (5):


FAA=Ln(Right(α))−Ln(Left(α)) (5)

#### 3.7.2 Data analysis

This study primarily used IBM SPSS Statistics 27 and MATLAB R2024b. First, all reverse-scored items on the FS-14 were inverted to facilitate subsequent analyses. We then conducted Shapiro–Wilk normality tests for the psychological scales (ROS, FS-14) across the four scenario groups (CGW, LGW, PGW, and NGW) as well as for the post-stressor measurements. As shown in Appendix Table 2, the *p*-values for both scales in each group were all greater than 0.05, indicating normality (Kim, 2013). To verify the stress-induction effect, we first performed independent-samples *t*-tests and, as needed, Levene’s tests for equality of variances: when *p* ≥ 0.05, we used the “equal variances assumed” *t*-test; when *p* < 0.05, we used the “equal variances not assumed” *t*-test, and in both cases we report the *p*-value from the *t*-test (Pernet et al., 2011). Next, before conducting between-group comparisons for the four scenarios (CGW, LGW, PGW, and NGW), we ran Levene’s tests separately for ROS and FS-14; results showed *p* = 0.052 for ROS and *p* = 0.281 for FS-14 (all *p* > 0.05), satisfying homogeneity of variance (Sawilowsky and Blair, 1992). Accordingly, we performed one-way ANOVA with “group” as a fixed factor and scale scores as the dependent variables. Overall tests indicated significant differences across the four groups for both scales (both *p* < 0.01), prompting *post hoc* pairwise comparisons using Bonferroni-adjusted p-values to determine significance (Shin et al., 2004). Effect sizes for pairwise contrasts were calculated and reported using Cliff’s Delta (*d*), where *d* > 0 indicates that the former group tends to be higher than the latter overall, and *d* < 0 indicates the opposite. Effect-size thresholds followed common cutoffs: |*d*| < 0.147 negligible, 0.147–0.330 small, 0.330–0.474 medium, and |*d*| ≥ 0.474 large (Romano et al., 2006; Santarém et al., 2023).

After conducting Shapiro–Wilk tests for all physiological indicators and EEG-based ratio indices, we found that the overall data did not meet the normality assumption (*p* < 0.05), as shown in Appendix Table 2. Therefore, for non-normally distributed EEG indices, we used the Kruskal–Wallis test to compare FAA and each power-ratio index across the four groups (Campos et al., 2025). When the omnibus test reached significance (*α* = 0.05), we performed pairwise comparisons using the Mann–Whitney *U* test, applied Bonferroni corrections to the pairwise *p*-values, and determined significance at adjusted *p* < 0.05 (Fong et al., 2010). Effect sizes for pairwise comparisons were calculated and reported using Cliff’s Delta.

## 4 Results

### 4.1 Evaluation of the stress-induction effect

**Figure 6:**
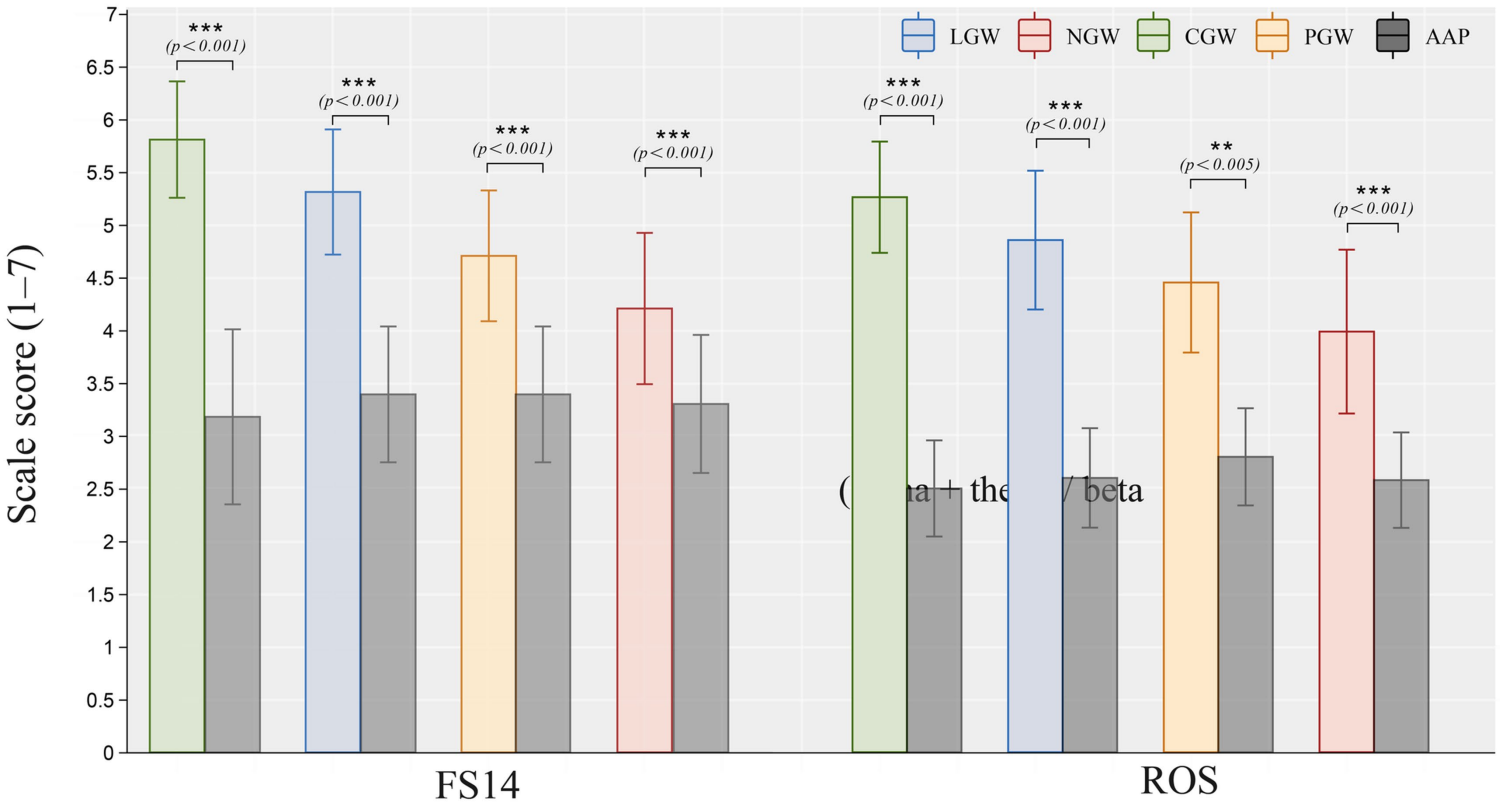
Independent-samples *t*-test results comparing post-stressor (AAP) subjective scales (FS-14, ROS) with all groups (CGW, LGW, PGW, NGW) (***, *p* < 0.001; **, *p* < 0.01; *, *p* < 0.05).

Figure 6 reports independent-samples t-tests comparing the post-stressor (AAP) subjective scales (FS-14, ROS) with all groups (CGW, LGW, PGW, and NGW). For the *t*-tests on the ROS questionnaire, Levene’s test was significant for the comparisons of LGW and NGW versus AAP (LGW: *p* = 0.032; NGW: *p* < 0.001); therefore, we report *t*-test results not assuming equal variances. In contrast, Levene’s tests were non-significant for CGW and PGW (CGW: *p* = 0.439; PGW: *p* = 0.207), and we accordingly report *t*-tests assuming equal variances. Statistical analyses showed that, on the ROS questionnaire, all four groups differed significantly from AAP (ΔM_CGW_ = 2.762, *p* < 0.001; ΔM_LGW_ = 2.255, *p* < 0.001; ΔM_PGW_ = 0.466, *p* < 0.005; ΔM_NGW_ = 1.408, *p* < 0.001), indicating that ROS scores in each group were significantly higher than AAP, as shown in Appendix Table 3. For the *t*-tests on the FS-14 questionnaire, Levene’s test was significant for CGW versus AAP (*p* = 0.020); therefore, we report *t*-test results not assuming equal variances. Levene’s tests were non-significant for LGW, PGW, and NGW (LGW: *p* = 0.598; PGW: *p* = 0.385; NGW: *p* = 0.203), and we accordingly report t-tests assuming equal variances. Statistical analyses showed that, on FS-14, all four groups differed significantly from AAP (ΔM_CGW_ = 2.623, *p* < 0.001; ΔM_LGW_ = 1.915, *p* < 0.001; ΔM_PGW_ = 1.588, *p* < 0.001; ΔM_NGW_ = 0.904, *p* < 0.001), indicating that FS-14 scores in each group were significantly higher than AAP, as shown in Appendix Table 3.

### 4.2 Subjective scale assessment

**Figure 7:**
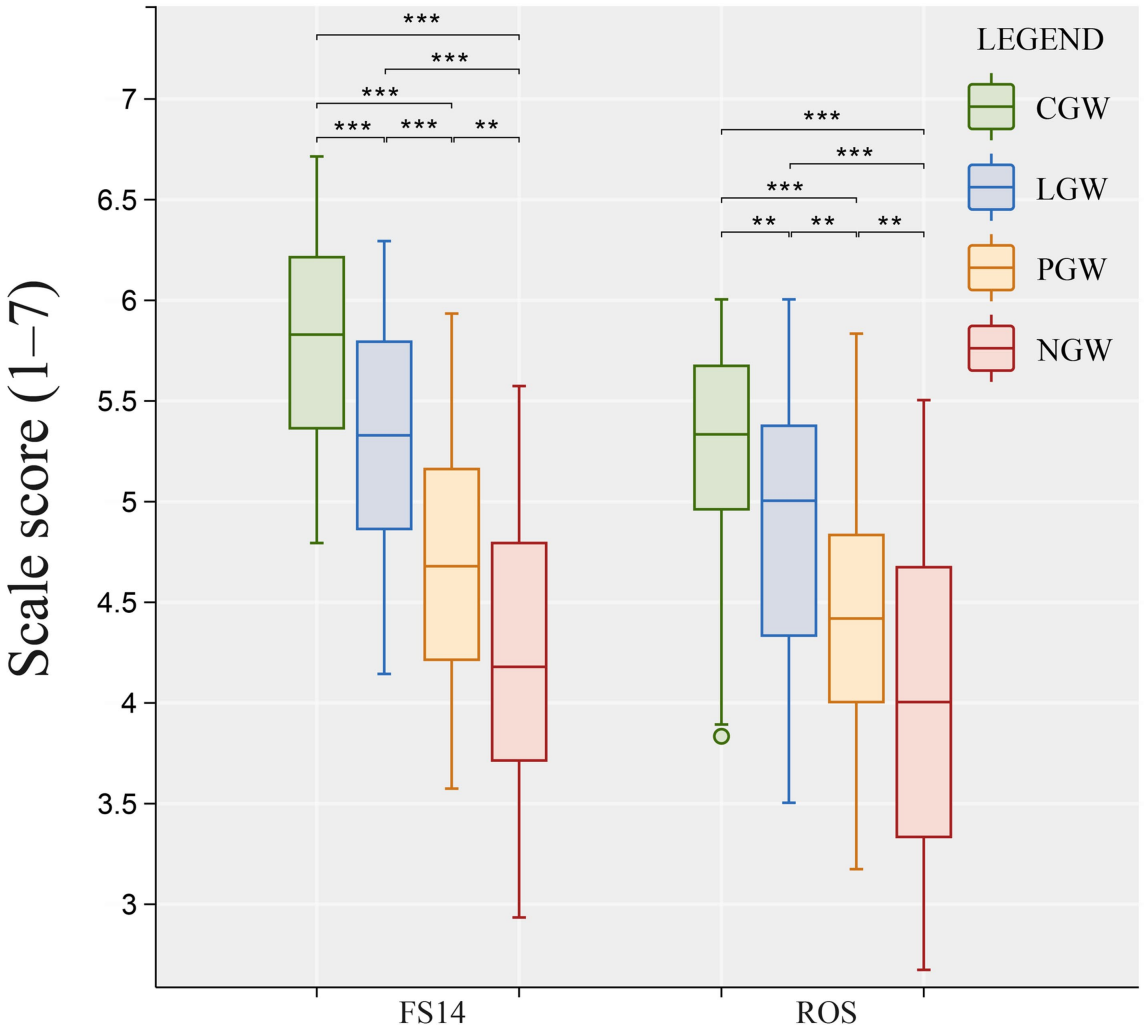
Effects of green-wall layouts on FS-14 and ROS scores (***, effect size = large; **, effect size = medium).

To compare differences in subjective alleviation across experimental groups, we conducted one-way ANOVAs on the subjective scales for each experimental material. As shown in Figure 7, the three experimental groups (CGW, LGW, and PGW) and the control group (NGW) exhibited significant differences on both the Fatigue Scale (FS-14) and the Restorative Outcome Scale (ROS). Specifically, the one-way ANOVA results were as follows: ROS [*F*(3, 156) = 27.137, *ω*^2^ = 0.329, *η*^2^ = 0.343, *p* < 0.01] and FS-14 [*F*(3, 156) = 50.24, *ω*^2^ = 0.48, *η*^2^ = 0.491, *p* < 0.01]. These results indicate that different green-wall layouts indeed produced significant differences in alleviating subjective fatigue and promoting restoration (Lakens, 2013). Further inspection of the group-wise data showed that all experimental groups (CGW, LGW, and PGW) performed significantly better than the control group (NGW) in terms of fatigue reduction and restorative effects, as shown in Table 1. Notably, in comparisons with the no–green wall control, the LGW group showed medium effect sizes on both FS-14 (*d* = 0.6457, *p* < 0.05) and ROS (*d* = 0.7448, *p* < 0.05). By contrast, the CGW group exhibited stronger subjective fatigue-recovery effects, showing medium effects only in its difference from the PGW group on ROS (*d* = 0.6839, *p* < 0.05), while all other contrasts displayed large effect sizes (all *p* < 0.001) (Stewart et al., 2022). These statistics suggest that, relative to an office without a green wall, green-wall designs effectively reduce subjective fatigue, with the CGW group demonstrating the strongest effects.

**Table 1:** Effect sizes and statistical significance of different green-wall layouts on FS-14 and ROS.

Scale	ANOVA test	Comparison	Cliff’s Delta	Bonferroni *p*-value	Effect size
ROS	*p* < 0.01	CGW vs. LGW	0.6839	*p* < 0.05	Medium
		CGW vs. PGW	1.3461	*p* < 0.001	Large
		CGW vs. NGW	1.9196	*p* < 0.001	Large
		LGW vs. PGW	0.6053	*p* < 0.05	Medium
		LGW vs. NGW	1.2047	*p* < 0.001	Large
		PGW vs. NGW	0.6457	*p* < 0.05	Medium
FS-14	*p* < 0.01	CGW vs. LGW	0.8673	*p* < 0.001	Large
		CGW vs. PGW	1.877	*p* < 0.001	Large
		CGW vs. NGW	2.5004	*p* < 0.001	Large
		LGW vs. PGW	0.9969	*p* < 0.01	Large
		LGW vs. NGW	1.677	*p* < 0.001	Large
		PGW vs. NGW	0.7448	*p* < 0.05	Medium

ROS ANOVA test: *F*(3, 156) = 27.14, *p* < 0.001, *η*^2^ = 0.343(*ω*^2^ = 0.33, Cohen’s *f* = 0.72).

FS14 ANOVA test: *F*(3, 156) = 50.24, *p* < 0.001, *η*^2^ = 0.491(*ω*^2^ = 0.48, Cohen’s *f* = 0.98).

### 4.3 Physiological response

#### 4.3.1 FAA results

We extracted alpha-band data from the left (AF3, F7, F3, and FC5) and right (AF4, F8, F4, and FC6) frontal channels for all four experimental conditions for FAA computation. The results showed that the median FAA values of the three experimental groups, CGW (Mdn = −0.020), LGW (Mdn = −0.010), and PGW (Mdn = −0.010), were all negative, whereas that of the control group NGW (Mdn = 0.020) was positive, indicating that green walls can promote increased right-side alpha power and bring individuals into an approach-related state. Subsequently, FAA values were examined using the Shapiro–Wilk test, and CGW, PGW, and NGW were found to deviate from normality, as shown in Appendix Table 2; therefore, a nonparametric Kruskal–Wallis test was employed to explore the differential effects of green-wall shapes on FAA. The two-sided Kruskal–Wallis test revealed significant differences among the four conditions [*χ*^2^(3) = 68.441, *p* < 0.01, *ε*^2^ = 0.449].

**Figure 8:**
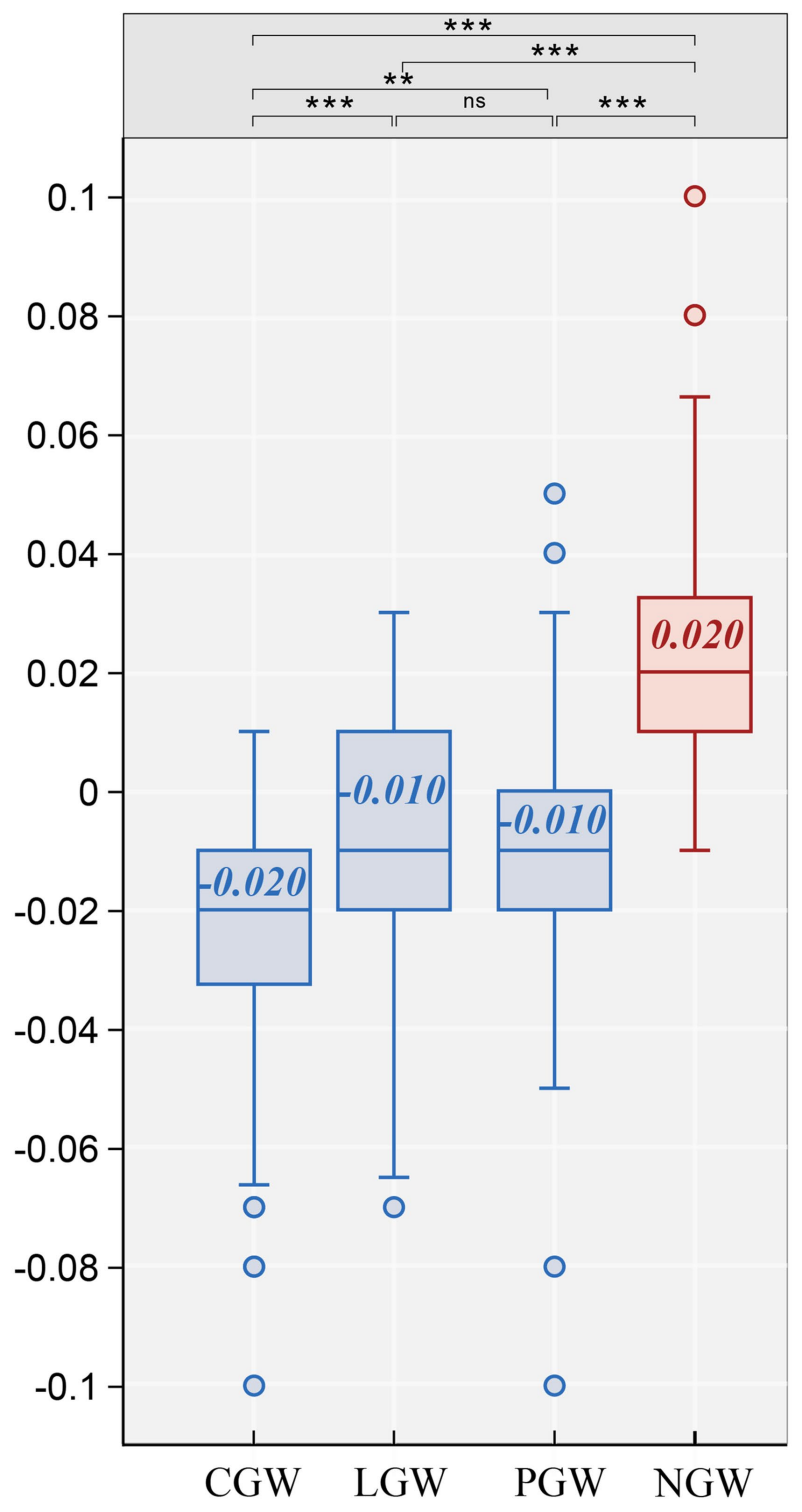
Between-group comparisons of the effects of different green-wall shapes on FAA (***, effect size = large; **, effect size = medium; ns, effect size = negligible; numbers denote medians).

As shown in Figure 8, *post hoc* tests indicated that, compared with the NGW group, all three green-wall groups had significantly lower FAA values, including the CGW group (*d* = −0.973, *p* < 0.001), LGW group (*d* = −0.688, *p* < 0.001), and PGW group (*d* = −0.728, *p* < 0.001), as reported in Table 2. This suggests that, relative to viewing a non-greened scene, viewing any type of green-wall scene can significantly elicit stronger approach motivation. Among the three green-wall groups, the CGW group showed significantly lower FAA values than the LGW group (*d* = −0.507, *p* < 0.005) and the PGW group (*d* = −0.469, *p* < 0.05), whereas no significant difference was found between LGW and PGW (*d* = 0.041, *p* > 0.05). Taken together, these findings indicate that the approach-related effect elicited by CGW is more pronounced than that produced by the generic green-wall design (NGW) and by PGW.

**Table 2:** Kruskal–Wallis tests and *post hoc* pairwise comparisons for FAA.

FAA	Kruskal–Wallis test	Comparison	Cliff’s *δ*	*p*-value	Effect size
FAA	*p* < 0.01	CGW vs. LGW	−0.507	*p* < 0.005	Large
		CGW vs. PGW	−0.469	*p* < 0.05	Medium
		CGW vs. NGW	−0.973	*p* < 0.001	Large
		LGW vs. PGW	0.041	*p*>0.05	Negligible
		LGW vs. NGW	−0.688	*p* < 0.001	Large
		PGW vs. NGW	−0.728	*p* < 0.001	Large

#### 4.3.2 Ratio performance

**Figure 9:**
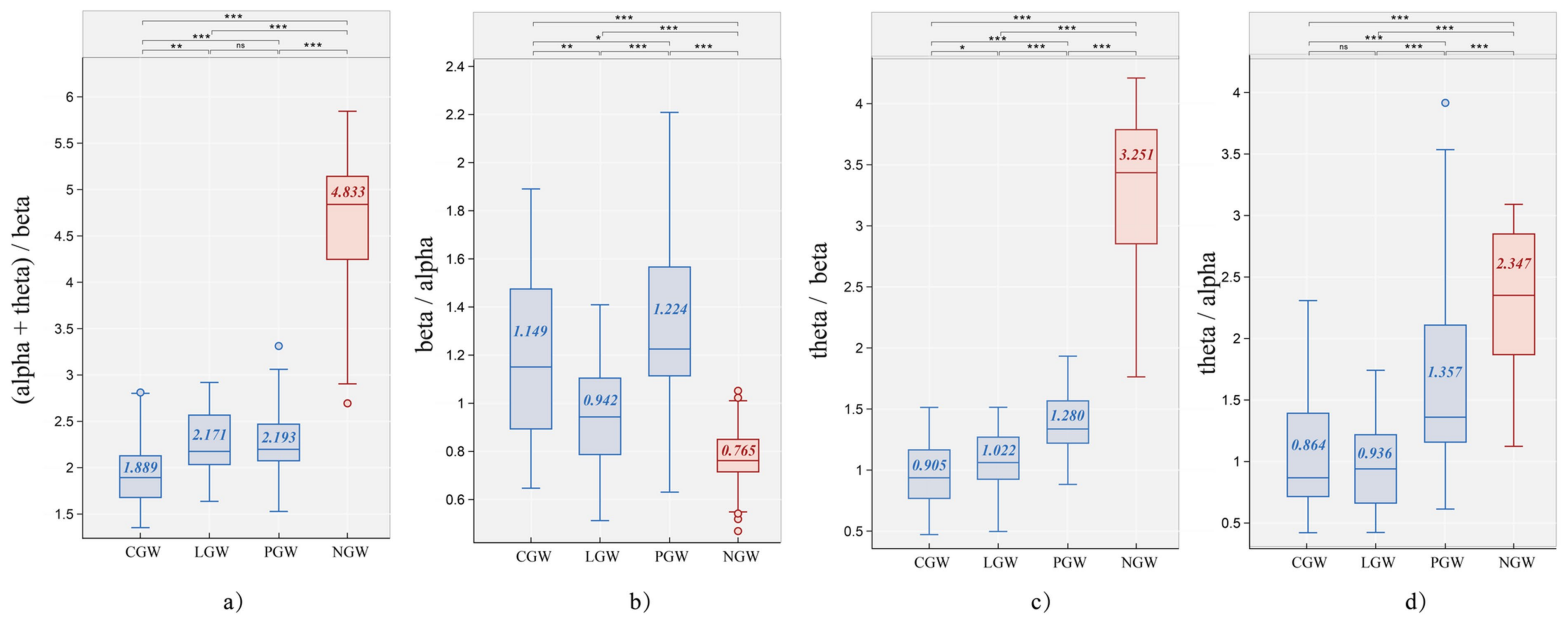
**(a)** Boxplot results of the EEG ratio (α+θ)/β for the four experimental groups (CGW, LGW, PGW, NGW) (***, effect size = large; **, effect size = medium; *, effect size = small; ns, effect size = negligible; numbers denote medians). **(b)** Boxplot results of the EEG ratio β∕α for the four experimental groups (CGW, LGW, PGW, NGW) (***, effect size = large; **, effect size = medium; *, effect size = small; ns, effect size = negligible; numbers denote medians).**(c)** Boxplot results of the EEG ratio θ∕β for the four experimental groups (CGW, LGW, PGW, NGW) (***, effect size = large; **, effect size = medium; *, effect size = small; ns, effect size = negligible; numbers denote medians). **(d)** Boxplot results of the EEG ratio θ∕α for the four experimental groups (CGW, LGW, PGW, NGW) (***, effect size = large; **, effect size = medium; *, effect size = small; ns, effect size = negligible; numbers denote medians).

According to the Kruskal–Wallis rank-sum tests, all four band-power ratios differed significantly across the four material types (all *p* < 0.01), Appendix Table 4. This indicates that the restorative effects induced by different green-wall materials are not identical. Further pairwise comparisons showed that NGW performed significantly worse than all three green-wall materials on every ratio (all *p* < 0.001; see Figure 9). To present the within-group differences among the three green-wall types—and their contrasts with the no–green wall condition—in greater detail, we report the statistics for each ratio separately below.

Figure 9a presents the analysis results for the fatigue index (α + θ)∕β (Table 3). The CGW group showed a more favorable fatigue-recovery effect than the PGW group (*d* = −0.495, *p* < 0.05) and the LGW group (*d* = −0.450, *p* = 0.05), while there was no significant difference between PGW and LGW (*d* = −0.035, *p* > 0.05). Relative to the NGW group, the fatigue index decreased by 60.91% in CGW, 55.08% in LGW, and 54.61% in PGW.

**Table 3:** Pairwise Mann–Whitney *U* comparisons of the four EEG ratios across the four groups (CCW, LGW, PGW, and NGW).

Ratio	*p*	Comparison	Cliff’s Delta	Effect size	*p*
(α + θ)/β	*p* < 0.01	CGW vs. LGW	−0.456	Medium	*p* = 0.05
		CGW vs. PGW	0.168	Large	*p* < 0.05
		CGW vs. NGW	−0.776	Large	*p* < 0.001
		LGW vs. PGW	0.634	Negligible	*p* > 0.05
		LGW vs. NGW	−0.485	Large	*p* < 0.001
		PGW vs. NGW	−0.850	Large	*p* < 0.001
β/α	*p* < 0.01	CGW vs. LGW	0.368	Medium	*p* < 0.05
		CGW vs. PGW	−0.178	Small	*p* > 0.05
		CGW vs. NGW	0.728	Large	*p* < 0.001
		LGW vs. PGW	−0.588	Large	*p* < 0.001
		LGW vs. NGW	0.490	Large	*p* < 0.05
		PGW vs. NGW	0.843	Large	*p* < 0.001
θ/β	*p* < 0.01	CGW vs. LGW	0.358	Small	*p* > 0.05
		CGW vs. PGW	−0.178	Large	*p* < 0.001
		CGW vs. NGW	0.728	Large	*p* < 0.001
		LGW vs. PGW	−0.588	Large	*p* < 0.005
		LGW vs. NGW	0.490	Large	*p* < 0.001
		PGW vs. NGW	0.843	Large	*p* < 0.001
θ/α	*p* < 0.01	CGW vs. LGW	0.095	Negligible	*p* > 0.05
		CGW vs. PGW	−0.529	Large	*p* < 0.001
		CGW vs. NGW	−0.900	Large	*p* < 0.001
		LGW vs. PGW	−0.626	Large	*p* < 0.001
		LGW vs. NGW	−0.954	Large	*p* < 0.001
		PGW vs. NGW	−0.549	Large	*p* < 0.005

Figure 9b reflects individual alertness and engagement/arousal. Unlike the results for (α + θ)∕β, on the β∕α index the PGW group showed a significant advantage over the LGW group (*d* = −0.588, *p* < 0.001), and its difference from the CGW group was a small, non-significant effect (*d* = −0.178, *p* > 0.05). Overall, PGW most effectively enhanced participants’ engagement and arousal, with a 60.00% increase relative to NGW. These results indicate that green-wall interventions can effectively elevate arousal levels, with the polyline layout performing best and the curvilinear layout approaching the polyline’s effect.

Figure 9c reports the θ∕β index used to characterize trait attentional control (Table 3). Compared with NGW, CGW exhibited a 72.16% reduction—the best among the three green-wall layouts; its difference from LGW was a small, non-significant effect (*d* = −0.265, *p* > 0.05). By contrast, PGW differed significantly from both CGW and LGW (all *p* < 0.001).

Figure 9d shows the θ∕α ratio used to reflect relaxation, where lower values indicate a more relaxed state. There was no significant difference between CGW and LGW (*d* = 0.095, *p* = 1.000); however, both showed significantly greater relaxation than PGW (all *p* < 0.001). Taken together, the latter two ratios suggest that, relative to the common green-wall design (LGW), the PGW tends to disperse attention, weaken control, and increase tension, whereas the curvilinear form more effectively facilitates relaxation while enhancing attentional level.

### 4.4 Correlation analysis

**Figure 10:**
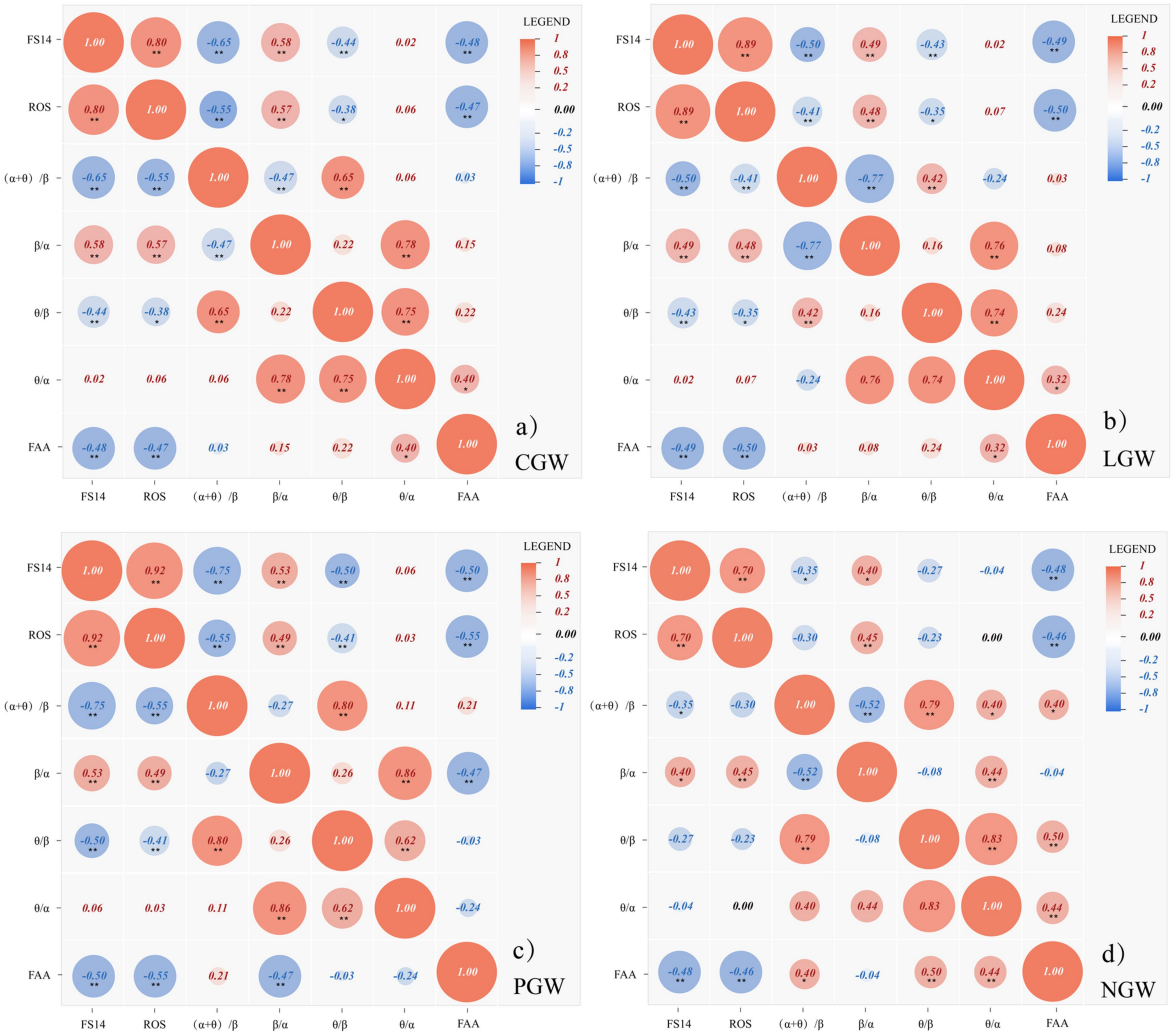
**(a)** Spearman correlation heatmap between the subjective scales (FS14, ROS) and EEG band-power ratios in the CGW group (**p < 0.01, *p < 0.05). **(b)** Spearman correlation heatmap between the subjective scales (FS14, ROS) and EEG band-power ratios in the LGW group (**p < 0.01, *p < 0.05). **(c)** Spearman correlation heatmap between the subjective scales (FS14, ROS) and EEG band-power ratios in the PGW group (**p < 0.01, *p < 0.05). **(d)** Spearman correlation heatmap between the subjective scales (FS14, ROS) and EEG band-power ratios in the NGW group (**p < 0.01, *p < 0.05).

Figure 10 presents the Spearman correlations among the subjective scales (FS14, ROS), frontal alpha asymmetry (FAA), and EEG band-power ratios under the four conditions (CGW, LGW, PGW, and NGW). Regarding the subjective scales, FS14 and ROS show significant positive correlations in all four groups (all *p* < 0.001), indicating that they jointly reflect an overall state of recovery and fatigue alleviation and that the effect of the experimental context on subjective restoration is stable across groups.

More specifically, in three of the experimental conditions, the correlations of FS14 and ROS with all band-power ratios except θ/α reach significance (all *p* < 0.01), and, as shown in Figure 10c, in the PGW group the correlations of FS14 with (α + θ)/β (*ρ* = −0.749, *p* < 0.001) and with θ/β (*ρ* = −0.498, *p* < 0.001) are the strongest in magnitude. Given that FS14 is a fatigue index after reverse coding, these negative correlations indicate that better subjective recovery is associated with lower physiological fatigue and better attentional control. This suggests that, within the PGW group, subjective recovery is consistently coupled with physiological fatigue and attention-related indices, and that the strength of this coupling is higher than in the other groups.

With respect to FAA, it shows significant correlations with both subjective questionnaires (all *p* < 0.01), indicating that when participants report better subjective fatigue relief and restoration, FAA tends to reflect an approach-related state. Furthermore, as shown in Figure 10, FAA is significantly correlated with θ/α (an index of relaxation) in the CGW, LGW, and NGW groups (all *p* < 0.05), whereas in the PGW group FAA shows a significant correlation with β/α (an index of arousal) (*p* < 0.05). In terms of correlations among band-power ratios, (α + θ)/β and θ/β exhibit strong positive correlations (*ρ* = 0.41–0.80, all *p* < 0.001), suggesting that in this experiment increased physiological fatigue is closely coupled with reduced attentional control. At the same time, θ/β and θ/α show a consistently strong association (*ρ* = 0.62–0.84, all *p* < 0.001), further indicating that in the present experimental context, attentional distraction and heightened tension or lack of relaxation tend to occur together, making individuals more likely to shift from a focused and stable working state to one of fatigue and distraction. Among the groups, the correlation between θ/β and θ/α is most pronounced in the CGW condition (Figure 10c), suggesting that under the curvilinear green-wall condition the co-occurrence of these two states is more synchronized and that a relaxed physiological state often accompanies focused attention. For the correlations between (α + θ)/β and β/α, most groups show significant negative correlations, indicating that increases in arousal are usually accompanied by reductions in fatigue; however, only in the PGW group does this relationship fail to reach significance (Figure 10d), suggesting that under the polyline green-wall condition the inverse coupling between arousal and fatigue is unstable and that arousal enhancement is not accompanied by a comparable reduction in fatigue as observed in the CGW and LGW groups.

## 5 Discussion

Most prior studies on indoor green walls have evaluated their impacts on psychological and physiological restoration from visual attributes such as plant combinations, color schemes, and coverage area. However, these studies have largely remained at the level of single visual attributes, with relatively few systematic examinations of how the combination of visual patterns and layouts integrated into a green wall modulates mind–body responses. Against this backdrop, the present study further tested the utility of green walls versus no green wall in office spaces at both psychological and physiological levels, and compared three typical layouts (curvilinear, linear, and polyline) in terms of promoting restoration and regulating alertness and attention.

After the literature review, this study proposed several core hypotheses, which are clearly addressed in the conclusion section. First, our results provide strong support for H1, namely that compared with NGW, all three green-wall layouts significantly promote fatigue recovery. Specifically, the presence of green walls led to marked improvements in scores on the ROS and on the reverse-coded FS-14, relative to both the post-stressor state and the NGW condition. At the same time, all four EEG ratio indices showed changes in the green-wall conditions that were favorable for restoration and regulation of alertness. Moreover, after participants experienced the three green-wall forms, FAA indicated a significantly stronger approach-related state compared with the NGW group and showed significant correlations with the subjective FS-14 and ROS scores. This suggests that exposure to green walls evoked approach-related motivational states and positive affect reflected in FAA, which is consistent with the findings of Ma et al. Taken together, these results not only support the core proposition of Attention Restoration Theory (ART), namely that green plants can restore the “directed attention” depleted by demanding tasks by strengthening people’s connection with nature in the environment (Ma et al., 2024; Raanaas et al., 2011), but also corroborate Stress Recovery Theory (SRT), which posits that natural elements exert effective stress-reducing effects by activating the parasympathetic nervous system and lowering physiological indices, thereby promoting relaxation, stress relief, and autonomic recovery. For example, Yin J. et al. (2020) compared biophilic and non-biophilic indoor environments in terms of heart rate, skin conductance, and self-rated anxiety, and their results consistently showed that biophilic elements produced more beneficial outcomes. The present study similarly demonstrates that green walls can serve as an effective carrier for both mechanisms, simultaneously supporting attentional restoration and emotional regulation within limited indoor space and ultimately yielding coordinated improvements in both subjective and physiological measures. However, unlike many previous studies, this work further examines how different layout designs of the same green-wall carrier still lead to distinct restorative benefits. In other words, the visual characteristics of green walls can shape the allocation of attentional resources and restoration-related mechanisms, thereby evoking differentiated physiological and psychological responses (Taylor, 2021; Vartanian et al., 2019). Based on these findings, conducting targeted analyses of between-group differences is necessary and can provide mechanism-based guidance for future green-wall design.

The second objective of this study was to examine how geometric form influences the directional tendency of green-wall benefits, and our experimental results show that the CGW group exhibited more favorable outcomes across all measurements in this experiment. In terms of the two subjective scales, the CGW group showed significantly better scores on subjective fatigue and restoration than both the control group and the other experimental groups, which is consistent with our hypothesis H2a. Regarding FAA, compared with the other groups, CGW, together with the high correlations observed between CGW and FAA, suggests that when participants experience better subjective recovery and reduced fatigue, this is accompanied by a stronger tendency toward active engagement (Harmon-Jones et al., 2010). In the EEG ratio results, CGW performed best on both (α + θ)/β and θ/α, with the former indicating lower physiological fatigue and the latter indicating a higher level of relaxation; this not only supports hypothesis H2b, but also provides a basis for further discussion on how CGW induces a relaxed state in participants. Moreover, on θ/β, which reflects attentional control, CGW was also significantly better than PGW and, together with LGW, fell within the most favorable range. Taken together, CGW showed the most beneficial effects in terms of physiological relaxation, fatigue recovery, and subjective experience. From a psychological perspective, Larson et al. (2007) noted that curvilinear forms can evoke associations with plant stems or with the raised cheeks and eyes in happy facial expressions, and such nature-based and positive-emotion associations foster pleasant feelings at the psychological level (Banaei et al., 2020). This is because such patterns are typically perceived as smooth and safe, and humans tend to show an innate preference for these fluent, nature-like forms (Blazhenkova and Kumar, 2018; Zhang et al., 2006). Ultimately, the consistency between this subjective preference and objective physiological responses was confirmed in our data: in the CGW group, scores on the subjective ROS and FS-14 scales showed highly significant correlations with multiple key EEG ratios, including (α + θ)/β, θ/β, and β/α. This not only allows for deeper discussion of emotion-related perception, but also highlights the gap that the present study helps to fill: prior research has mostly compared forms to explore how they shape subjective impressions and visual preferences, whereas our findings show that such subjective visual preferences concretely modulate the direction and magnitude of benefits produced by biophilic design. To further examine how curves influence attention and relaxation, we draw on key insights from an eye-tracking study by Biswas et al., which showed that the advantage of curvilinear forms does not lie in attracting initial fixations faster than sharp-angled forms, but rather in retaining attention once they enter the visual field, as reflected in longer total gaze duration and a greater number of fixations (Biswas et al., 2024). This helps to explain why, on β/α as an index of arousal, CGW, although slightly lower than PGW, still exhibits a clear arousal and engagement effect. At the same time, prolonged viewing of preferred patterns often leads individuals into a relaxed state, because such patterns provide a more comfortable visual environment, which in turn explains the significant association observed between high attention and high relaxation under the CGW condition.

Compared with the other groups, PGW showed poorer subjective fatigue and recovery on FS14 and ROS; however, it is noteworthy that the PGW group exhibited a more pronounced response on β/α, a widely used index of arousal, indicating that beta activity was enhanced and alpha activity was relatively suppressed, which reflects an elevated level of arousal (Spironelli and Borella, 2021). In terms of FAA, there was no significant difference between LGW and PGW, and both showed negative median values (stronger left frontal activity), suggesting that participants were in an approach-related state; combined with the correlation analysis, FAA showed strong negative correlations with both subjective ratings and β/α (*p* < 0.05), indicating that an approach-related FAA pattern tends to co-occur with higher arousal and lower perceived fatigue recovery, which may stem from motivational responses to discomfort, conflict, or stress (Verona et al., 2009). In other words, when the subjective ratings are considered together with the FAA results, it becomes evident that although the polyline form does not foster much relaxation, it does possess an “attention-grabbing” or “stimulating” quality (Suh and Yim, 2018; Hsu and Wang, 2013), which is consistent with our hypothesis H2c. Moreover, this view is supported by Larson et al. (2007), who showed that V-shaped or polyline configurations, due to their structural discontinuity, can more effectively disrupt visual regularity and therefore rapidly capture observers’ attention. However, the cost of this attention capture is its potentially negative emotional valence, since sharp V-shaped contours are easily associated with features such as the eyebrows or jawline in angry facial expressions, thereby implicitly conveying a sense of tension or even confrontation, which helps explain why the polyline green wall produced strong arousal but relatively poor emotional and subjective outcomes. Within the context of plant forms, this perspective is further supported by Hareli et al. (2016), who argued that sharp plant leaves, because of their evolutionary role as “anti-herbivore defense,” tend to be subconsciously perceived by humans as more dangerous and threatening, thereby eliciting heightened vigilance and unease. Further analysis showed that in the PGW condition, β/α was strongly correlated with θ/α, θ/β, and (α + θ)/β, with correlation strengths generally higher than in the other groups; among the patterns shared by all three green-wall groups, θ/β and θ/α were also highly correlated, pointing to a possible chain in which high engagement and high arousal do not equate to relaxation and may instead be accompanied by constrained allocation of attentional resources and increased fatigue. To explore the underlying reasons, we noted that the polyline form rapidly increases vigilance after stress induction, and such short-term activation is likely to trigger “stress-related alertness” driven by subjective discomfort (Richards et al., 2014). This subjectively induced alertness, whether caused by negative feelings or unpleasant images, directly modulates EEG activity, particularly high-frequency beta oscillations in frontal and parietal regions (Olofsson et al., 2008; Güntekin and Tülay, 2014), which in turn helps explain the significant positive correlation observed between the β/α ratio and the subjective recovery score FS14. Overall, although PGW performed worse than CGW and LGW on most restorative indices, it seems to offer a recovery pathway characterized by high arousal; that is, while its overall restorative effect is inferior to that of the other green-wall groups, it provides a distinct “high-arousal” recovery route, since its final outcomes still significantly surpass those of the no–green wall control. In addition to psychological factors, sustained high attentional focus after stress induction may, over time, impose mental and physical burdens on participants and increase fatigue, which may also contribute to the observed results; therefore, it is necessary to take the influence of exposure duration into account (Van Cutsem et al., 2017).

As a green wall with a linear configuration, LGW to some extent represents one of the most common contemporary green-wall styles. In this experiment, the subjective measures (FS-14, ROS) showed that the LGW group achieved relatively favorable subjective fatigue recovery. Further pairwise comparisons indicated that, in terms of arousal level, the LGW group was significantly weaker than the other two green-wall forms, whereas its physiological fatigue level did not differ significantly from that of the PGW group (*p* > 0.05). This implies that, compared with polyline and curvilinear forms, a conventional linear green wall is not particularly effective in enhancing arousal, and after stress induction it also fails to match CGW in terms of fatigue recovery and attentional control. By comparison, Li et al. (2025) found that rectangular green walls (including square configurations) are more advantageous than triangular ones in relieving anxiety and reducing attentional resource depletion, and part of their results are consistent with the trends observed in this study. However, this study also included a curvilinear form in the comparison, and the results showed that hypothesis H2d was not supported. Although the findings suggest that LGW and CGW differ only slightly in attentional control, it is still necessary to further examine the reasons behind this and to reconsider the linear form as a design variable in its own right. Previous research has shown that the edges and corners of rectangles do not have an advantage in attracting initial fixations, and once they enter the visual field they are less able to sustain interest, with attention quickly decaying and habituation occurring, which leads to only limited increases in arousal (Biswas et al., 2024); this helps to explain why LGW produced lower arousal levels than the other groups. In addition, as one of the most common green-wall styles in everyday environments, such a design may struggle to offset feelings of irritability and other negative emotions once individuals are under stress (Fredrickson and Branigan, 2005; Guy et al., 2023), because people find it difficult to maintain prolonged gaze and deep relaxation in response to such highly familiar forms (Tinio and Leder, 2009). Moreover, Shemesh et al. (2021) further demonstrated that simple symmetric shapes such as squares, because they are overly familiar and perceptually fluent, are often associated with lower arousal levels and lack sufficient complexity of neural stimulation, which limits their potential to optimize the allocation of brain resources.

## 6 Conclusion

This study used virtual reality (VR) to compare the effects of different green-wall geometries (CGW, PGW, LGW) on psychological and physiological restoration in office workers after stress induction. The main conclusions are as follows:

(1) Compared with NGW in post-stress recovery, all forms of green walls effectively improved participants’ psychological and physiological health, and hypothesis H1 was supported. Specifically, all green-wall groups showed clear advantages over NGW in subjective fatigue tests, restorative ratings, and EEG ratio indicators. However, it is noteworthy that there were differences in effect size among the three geometries, indicating that visual form is an important variable influencing mind–body responses.
(2) Compared with LGW and PGW, the CGW group showed the most positive subjective recovery, which confirms H2a. In terms of physiological indicators, CGW also performed best on the fatigue index, which supports H2b. Furthermore, CGW exhibited more favorable median levels in attention and relaxation. Although the differences between CGW and LGW in attention and relaxation did not reach statistical significance, these results suggest that the curvilinear green wall not only maintains the general restorative benefits of green walls but also provides superior performance in alleviating fatigue, supporting attentional control, and enhancing relaxation.
(3) The PGW group did not differ significantly from LGW in fatigue level, but it was significantly worse than the other groups in attention recovery and relaxation. However, for the arousal index, the difference between PGW and CGW was small, and both groups were significantly better than LGW, which provides solid support for H2c. This pattern indicates that although the polyline form is weaker on restorative indicators, it is more effective at increasing arousal in office users.

Although this study makes several contributions, there are still some limitations. First, our discussion suggests that green walls can enhance attention, but maintaining heightened attention over a long period may consume the short-term benefits or even lead to negative effects, which appears particularly evident in the polyline condition. Therefore, future research should introduce exposure duration as a factor and examine dose–response relationships across different viewing times. In addition, this study adopted several EEG band-power ratios as physiological indices. This approach effectively integrates individual differences in single-band EEG power and provides high sensitivity in short-term environmental exposures, which aligns well with our “post-stress short-term experience” design. However, it also means that the study lacks detailed analysis of single-band power, electroencephalographic topography, and other EEG measures. Future work should therefore consider incorporating more fine-grained frequency and spatial indicators in order to build a more rigorous measurement framework. Third, participants were required to wear both EEG equipment and a VR headset during the experiment, which may have reduced comfort and increased burden, thereby influencing subjective and physiological responses. Due to constraints of currently available devices, future studies could consider designs in which participants wear only EEG, only VR, or view non-immersive displays, and then compare the consistency of results across different recording modes. Finally, in order to make VR exposure manageable for participants, the sample size was limited to 40 people per group across the four conditions, which may restrict our ability to detect subtle effects. Future studies can increase the sample size to improve statistical power and external validity, thereby yielding more comprehensive and generalizable conclusions.

Overall, the presence of green walls can effectively improve the experiential quality of office environments, and, more specifically, different geometric forms show distinct strengths in the patterns of subjective and physiological responses they elicit. These findings provide more targeted guidance for selecting facade geometries in interior design and help to link form, planting, and mind–body responses into a coherent conceptual chain, outlining promising directions and prospects for future applications and studies in diverse tasks and settings.

## Data Availability

The dataset can be provided upon reasonable request by contacting the corresponding author.
