# Supplementary material for: Effects of green-wall layouts on psychological and physiological responses in office environments: a virtual-environment study
Source: Front Psychol. 2026 Jan 12;16:1711317. doi: 10.3389/fpsyg.2025.1711317 (PMC12832635; doi:10.3389/fpsyg.2025.1711317)
Supplement: Supplementary file 1 [file Supplementary_file_1.docx]

Supplementary Material

# Supplementary Figures and Tables

| Scale | | Question |
| --- | --- | --- |
| ROS | Q1 | I feel calm. |
|  | Q2 | I feel more focused and alert. |
|  | Q3 | I feel a renewed enthusiasm and energy for daily life. |
|  | Q4 | I feel restored and relaxed. |
|  | Q5 | I can forget my everyday worries. |
|  | Q6 | I feel better able to organize my thoughts and ideas. |
| FS-14 | Q1 | Do you feel fatigued? |
|  | Q2 | Do you feel you need more rest? |
|  | Q3 | Do you feel drowsy or constantly sleepy? |
|  | Q4 | Do you find it difficult to get started on anything? |
|  | Q5 | Can you start tasks easily but quickly become tired over time? |
|  | Q6 | Do you feel low in energy or lacking vitality? |
|  | Q7 | Do you feel your muscular strength is decreasing? |
|  | Q8 | Do you feel physically weak? |
|  | Q9 | Do you have difficulty concentrating? |
|  | Q10 | Do you feel mentally unclear or sluggish in thinking? |
|  | Q11 | Do you frequently make slips of the tongue? |
|  | Q12 | Do you find it hard to come up with the right words to express yourself? |
|  | Q13 | Do you feel your memory has worsened? |
|  | Q14 | Have you lost interest in things you used to enjoy? |

## **Appendix Table 1.** Item lists for the Restorative Outcome Scale (ROS) and the Fatigue Scale (FS-14).

| Psychological Data — Shapiro–Wilk | | | |
| --- | --- | --- | --- |
| Measure | Group | Shapiro–Wilk W | p-value (> 0.05) |
| ROS | c1 | 0.951 | 0.084 |
|  | c2 | 0.961 | 0.183 |
|  | c3 | 0.979 | 0.668 |
|  | c4 | 0.963 | 0.215 |
| FS-14 | c1 | 0.96 | 0.171 |
|  | c2 | 0.97 | 0.364 |
|  | c3 | 0.976 | 0.541 |
|  | c4 | 0.973 | 0.439 |
| ROS（After applying pressure） | c1 | 0.97 | 0.369 |
|  | c2 | 0.954 | 0.102 |
|  | c3 | 0.966 | 0.274 |
|  | c4 | 0.96 | 0.171 |
| FS-14（After applying pressure） | c1 | 0.968 | 0.305 |
|  | c2 | 0.97 | 0.351 |
|  | c3 | 0.97 | 0.369 |
|  | c4 | 0.969 | 0.337 |
| Physiological Data — Shapiro–Wilk | | | |
| Section | EEG Ratio | Group | Shapiro–Wilk W |
| (α+θ)/β | c1 | 0.971 | 0.374 |
| (α+θ)/β | c2 | 0.957 | 0.129 |
| (α+θ)/β | c3 | 0.959 | 0.156 |
| (α+θ)/β | c4 | 0.93 | 0.016 |
| β/α | c1 | 0.946 | 0.055 |
| β/α | c2 | 0.98 | 0.705 |
| β/α | c3 | 0.969 | 0.342 |
| β/α | c4 | 0.977 | 0.582 |
| θ/β | c1 | 0.912 | 0.004 |
| θ/β | c2 | 0.957 | 0.131 |
| θ/β | c3 | 0.87 | 0 |
| θ/β | c4 | 0.944 | 0.046 |
| θ/α | c1 | 0.912 | 0.004 |
| θ/α | c2 | 0.957 | 0.131 |
| θ/α | c3 | 0.87 | 0 |
| θ/α | c4 | 0.944 | 0.046 |

**Appendix Table 2.** Shapiro–Wilk normality test results for physiological and psychological data

| Questionnaire | Comparison | Levene’s test (p > 0.05) | Significance (two-tailed) | Mean difference | 95% CI of the difference | |
| --- | --- | --- | --- | --- | --- | --- |
|  |  |  |  |  | Lower | Upper |
| ros | CGW vs AAP | 0.439 | p＜0.001 | 2.762 | 2.542 | 2.98099 |
|  | LGW vs AAP | 0.032 | p＜0.001 | 2.255 | 2.000 | 2.50982 |
|  | PGW vs AAP | 0.207 | p＜0.001 | 0.466 | 0.144 | 0.78721 |
|  | NGW vs AAP | 0 | p＜0.001 | 1.408 | 1.124 | 1.69242 |
| fs-14 | CGW vs AAP | 0.02 | p＜0.001 | 2.623 | 2.309 | 2.93725 |
|  | LGW vs AAP | 0.598 | p＜0.001 | 1.915 | 1.640 | 2.19016 |
|  | PGW vs AAP | 0.385 | p＜0.001 | 1.588 | 1.292 | 1.88343 |
|  | NGW vs AAP | 0.203 | p＜0.001 | 0.904 | 0.598 | 1.20863 |

**Appendix Table 3.** Independent-samples t-tests: AAP vs Groups (ROS & FS-14).

| Content | EEG Ratio | Result |
| --- | --- | --- |
| ε² | （α+θ）/β | 0.614 |
|  | β/α | 0.358 |
|  | θ/β | 0.699 |
|  | θ/α | 0.493 |
| χ²/df | （α+θ）/β | 98.822/3 |
|  | β/α | 58.662/3 |
|  | θ/β | 112.069/3 |
|  | θ/α | 79.936/3 |

**Appendix Table 4.** Overall effect sizes and degrees of freedom for the four experimental groups (CCW, LGW, PGW, NGW) across the four EEG ratios
